# A Roadmap for the Development of Ivermectin as a Complementary Malaria Vector Control Tool

**DOI:** 10.4269/ajtmh.19-0620

**Published:** 2020-02-06

**Authors:** The Ivermectin Roadmappers

**Affiliations:** 1 Full List of Contributors in the Acknowledgments

## Abstract

In the context of stalling progress against malaria, resistance of mosquitoes to insecticides, and residual transmission, mass drug administration (MDA) of ivermectin, an endectocide used for neglected tropical diseases (NTDs), has emerged as a promising complementary vector control method. Ivermectin reduces the life span of *Anopheles* mosquitoes that feed on treated humans and/or livestock, potentially decreasing malaria parasite transmission when administered at the community level. Following the publication by WHO of the preferred product characteristics for endectocides as vector control tools, this roadmap provides a comprehensive view of processes needed to make ivermectin available as a vector control tool by 2024 with a completely novel mechanism of action. The roadmap covers various aspects, which include 1) the definition of optimal dosage/regimens for ivermectin MDA in both humans and livestock, 2) the risk of resistance to the drug and environmental impact, 3) ethical issues, 4) political and community engagement, 5) translation of evidence into policy, and 6) operational aspects of large-scale deployment of the drug, all in the context of a drug given as a prevention tool acting at the community level. The roadmap reflects the insights of a multidisciplinary group of global health experts who worked together to elucidate the path to inclusion of ivermectin in the toolbox against malaria, to address residual transmission, counteract insecticide resistance, and contribute to the end of this deadly disease.

## INTRODUCTION

### Malaria situation.

Malaria remains a significant public health problem worldwide, particularly across low- and middle-income regions. Although the disease is both preventable and curable, it currently threatens nearly half of the world’s population living in 90 malaria-endemic countries.^1^ Over the last two decades, the large-scale implementation of preventive strategies, as well as the improvements in the diagnosis and treatment of the disease, has led to an 18% global drop in incidence rates between 2010 and 2017.^1^ Vector control with insecticide-treated nets (ITNs) and indoor residual spraying (IRS) is considered the main driver of these malaria gains (i.e., ITNs and IRS accounted for 78% of the malaria cases averted between 2000 and 2015).^2^ Despite these advances, in 2017, there were 219 million cases and 435,000 malaria deaths estimated globally, with 93% of deaths occurring in Africa.^1^ After over a decade of downward trends, the 2017 and 2018 World malaria reports have shown that progress has stalled, especially in high-burden countries. This poses a great challenge in achieving the morbidity and mortality targets of the WHO Global Technical Strategy (GTS) for Malaria 2016–2030.^1,3^

Among the challenges currently weakening vector control are mosquito resistance to insecticides and residual transmission. Residual transmission is defined as the persistence of malaria transmission after universal coverage with effective ITNs and/or IRS to which the local vectors are fully susceptible.^4^ Through behavioral adaptations, mosquitoes are able to avoid the standard vector control measurements by biting while humans are not protected by ITNs and/or outdoor, as well as feeding on peri-domestic livestock. Thus, research and product development are critical to mitigate the existing protection gaps. Along these lines, the GTS^5^ and the research agenda for malaria control proposed by the malERA consultative group^6^ reflect the need for improved ITNs and innovative ways of controlling residual transmission.

### The potential role of ivermectin as a complementary vector control tool against malaria.

Ivermectin is a long-established veterinary endectocide, first approved for human use for its antiparasitic activity against onchocerciasis in 1987.^7^ In 2018, global health authorities celebrated 30 years of ivermectin mass drug administration (MDA) campaigns against two neglected tropical diseases (NTDs), onchocerciasis, and lymphatic filariasis (LF).^8^ Besides its broad antiparasitic activity, ivermectin can kill mosquitoes that feed on treated humans and livestock during a dose-dependent period.^9–12^ This occurs because ivermectin binds selectively to the glutamate-gated chlorine channel of invertebrates and produces paralysis.^7^ By exploiting this mosquitocidal activity, ivermectin MDA to humans and/or livestock could complement the malaria toolbox, reducing mosquito survival regardless of their biting patterns.

The notion of repurposing ivermectin for malaria control originally emerged in 1985 when in vitro tests showed that the drug killed the malaria vector *Anopheles stephensi.*^13^ Additional data appeared slowly thereafter. A study in Papua New Guinea in 1999 demonstrated that even a single-standard dose of ivermectin affected vector survival in the field.^14^ A randomized controlled trial further supported the killing effect on mosquitoes feeding on treated people.^15,16^ More recently, the results of several modeling, pharmacological, and insectary-based research studies, as well as several clinical and field MDA trials, have positioned ivermectin as a first-in-class tool to enhance malaria control.^17–19^

After recognizing the potential for endectocides to tackle the issue of residual transmission, the WHO held a technical consultation on ivermectin in 2016. Subsequently, preferred product characteristics (PPCs) for endectocides against malaria were published with ivermectin as a reference product.^20^ The meeting report, endorsed by the Malaria Policy Advisory Committee (MPAC), also established the requirement for a WHO policy recommendation on ivermectin for malaria control. This requirement is that a minimum 20% decrease in malaria incidence should be achieved for at least 1 month posttreatment after a single round of ivermectin MDA when added to the standard vector control tools.^20^ Given this momentum, several funders began supporting the evaluation of endectocides against malaria, reflecting the new guidance and broad interest in this innovative approach (current and planned trials are discussed in section Ongoing/planned trials).

### The malaria Ivermectin Roadmap.

The objective of the Ivermectin Roadmap is to define a clear pathway for the evaluation of ivermectin as a vector control tool against malaria and for its subsequent implementation. Specifically, this analysis has carefully considered: 1) product development (e.g., dose and regimen), 2) evidence to support a global policy recommendation (e.g., safety and efficacy), and 3) access and deployment at scale (e.g., procurement and delivery mode). Throughout the different sections of this article, key research and development (R&D) questions have been identified so as to outline an R and D agenda for the development of ivermectin as a complementary vector control tool.

Over the past 4 years, experts in relevant fields have defined the critical aspects of repurposing ivermectin as a complement to current malaria vector control tools. In 2014, the Ivermectin Research for Malaria Elimination Network,^21^ began shaping the concept. The Ivermectin Roadmappers were assembled in 2017 after funding was granted by the Bill & Melinda Gates Foundation. A launch meeting took place at the 2017 Annual Meeting of the American Society of Tropical Medicine and Hygiene (ASTMH) in Baltimore (US). The multidisciplinary team included global health experts whose backgrounds encompassed entomology, infectious diseases, vaccines, veterinary, environmental sciences, ethics, financing systems, clinical trials, supply chain management, and scaling-up of interventions, among others (see full list of contributors on pages 18–19).

The process to develop this roadmap consisted of literature reviews by the participants, as well as discussions with regulatory agencies, policy and funding bodies, drug manufacturers, and future implementing partners, including the WHO and Unitaid. A synthesis meeting was held in Sitges (Spain) in May 2018, where key aspects were discussed and refined. The outcomes of the gathering were publicly presented during the symposium “*A Roadmap for Ivermectin as a Complementary Vector Control Tool for Malaria*” at the 2018 ASTMH Annual Meeting (https://mesamalaria.org/resource-hub/astmh-2018-session-30-roadmap-ivermectin-complementary-vector-control-tool-malaria) and are now reflected in this roadmap.

## THE GOAL

### Vision.

To accelerate global malaria control and elimination with a novel vector control tool that addresses residual transmission and mitigates the risk of insecticide resistance.

### Strategic goal.

To advance a complementary strategy for vector control that reduces malaria burden (incidence) by at least 20% when deployed at the community level in addition to ITNs and/or IRS.

## PRODUCT DEVELOPMENT NEEDED TO REPURPOSE IVERMECTIN TO MALARIA

### Use scenarios.

The rationale for an ivermectin-based approach against malaria is that it reduces the longevity of mosquitoes that feed on ivermectin-treated subjects. The effect is dose-dependent. Thus, deploying ivermectin to an important (or significant) proportion of humans and/or the predominant herd during the malaria season could significantly reduce transmission of the disease. Moreover, the resulting decline in mosquito populations can boost the effects of core vector control tools (i.e., ITNs or IRS), leading to an overall higher impact. The community delivery of ivermectin has the potential to fill an important gap in vector control by addressing residual transmission. Residual transmission is driven by mosquitoes biting outdoors and/or early in the evening, mosquitoes feeding on peri-domestic livestock, and human behaviors that decrease the effectiveness of current vector control programs. In addition to tackling residual transmission, ivermectin belongs to a different chemical class than the active ingredients present in ITNs or sprays, potentially contributing to insecticide resistance management. Such properties place ivermectin MDA as an attractive addition to the malaria control toolbox. Other MDA strategies with blood and/or tissue schizonticidal drugs address the parasite biomass circulating in the human population and do not impact the vector or the parasites they may be carrying. However, before deployment, key questions regarding the delivery of this drug will need to be answered, including the determination of the range of effective and safe doses, the target population, the required level of community uptake, the malaria epidemiologic context, and the distribution strategies, among others.

Given the considerable effort associated with the high-level community delivery of a relatively short-acting drug, the use of ivermectin could be suitable for short and intense use, as opposed to the more enduring use of other measures such as ITNs.^20^ There are different potential approaches to the implementation of ivermectin MDA as vector control:

1. Ivermectin alone to complement the national strategy of deploying ITNs and IRS.
2. Ivermectin MDA combined with antimalarial drugs to simultaneously clear infections in humans, provide time-limited chemoprevention, and prevent transmission, increasing impact.
3. Ivermectin co-administered at the same time as seasonal malaria chemoprevention (SMC) programs in areas with a short transmission season. Although the target populations of SMC and ivermectin MDA differ, the latter could benefit from the current door-to-door delivery strategy of SMC to children in households, to also deliver ivermectin to the rest of the eligible population.
4. In parallel, in many but not all areas where malaria ivermectin MDA would be distributed, there may exist the opportunity to create synergies with the national NTD treatment programs because twice a year, ivermectin is often recommended for onchocerciasis and for LF eradication. The need to deliver as part of a combination regimen for LF, varying dosages across programs, and different sources of ivermectin need to be rationalized where joint programming is considered.

Taking the aforementioned key aspects into account, Table 1 summarizes potential use scenarios for ivermectin as vector control. Notably, the first four scenarios will be tested in various trials through 2023.

**Table 1:** Potential use scenarios for ivermectin in different transmission settings, delivered to different target blood sources, and under several co-delivery models

Transmission setting	Rationale for ivermectin use	Target blood source	Always present	Additional co-delivery	Rationale for co-delivery
Higher	Reduce disease burden	Human	As per national policy: ITNs or IRS Case management IPTp	SMC	Using SMC as a platform for ivermectin delivery, operational synergism is achieved
Higher	Accelerate to elimination	Human		ACT MDA	Ivermectin provides additional transmission reduction by targeting outdoor and early biting vectors
Higher	Reduce vectorial capacity	Livestock		Behavior change interventions to boost ITN use and treatment of cases	Protect households and drive vectors to zoophagy; this strategy allows the use of long-lasting veterinary formulations
Higher	Reduce vectorial capacity	Human + livestock		With or without ACT MDA	Covering different blood sources could increase impact on local vector populations
Higher	Reduce vectorial capacity	Human		IRS timed after ivermectin MDA	Improve IRS efficacy by precipitating a sharp reduction in vectors right before the IRS campaign
Any	Reduce disease burden Reduce vectorial capacity	Human		NTD interventions such as azithromycin or IDA for lymphatic filariasis	As part of joint efforts with NTD programs
Any	Insecticide resistance management	Human ± livestock		PBO and next-generation nets, other insecticide delivery vehicles, i.e., attractive targeted sugar baits	As part of an insecticide resistance management strategy
Lower	As part of reactive interventions	Human ± livestock		As part of focal MDA with ACT ± other vector control tools	Prevention of secondary cases at low transmission levels
Any	Prevent or manage outbreaks	Human ± livestock		MDA with ACT + ivermectin ± other vector control tools	As a way to quickly reduce vectorial capacity

IDA = triple therapy with ivermectin, diethylcarbamazine and albendazole; IPTp = intermittent preventive treatment in pregnancy; IRS = indoor residual spraying; ITN = insecticide-treated net; MDA = mass drug administration; NTD = neglected tropical disease; SMC = seasonal malaria chemoprevention

#### Target population.

Ivermectin MDA is a vector control intervention designed to be administered to humans to affect the survival of mosquitoes that feed on them. In addition, farm animal ownership has been described as a risk factor for human malaria infection in areas where mosquitoes feed on both animals and humans; thus, the administration of ivermectin to both sources of blood has been proposed.^22–25^ Conveniently, ivermectin is an antiparasitic drug already broadly used in human and veterinary contexts to treat a range of endo- and ectoparasites.^26^ Ivermectin MDA would target mosquitoes feeding on treated subjects (humans or animals) regardless of their biting patterns. A key concept is coverage threshold for efficacy based on the target population. In human populations, modeling suggests that coverage as low as 60% could have a significant impact on transmission,^27,28^ but no model has yet explored the impact threshold for targeting livestock and humans together. Although the advantages and impact of treating humans only, livestock only, or both need to be assessed in field studies, covering the two sources of mosquito meals could result in major impact on human health, especially in settings where the principal malaria vector species exploit both as sources of blood meals.

#### Malaria endemicity.

The introduction of ivermectin to suppress vectorial capacity could be beneficial in a range of malaria transmission intensities. This report considered the value of ivermectin as a complementary tool to achieve impact in high-transmission areas, as well as serving as a short-term adjunct to achieving elimination in lower incidence areas. In such settings, ivermectin MDA would indirectly target the mosquitoes, in contrast to MDA with antimalarials in which the aim is to decrease malaria burden by directly attacking the human reservoir for the parasite. However, the combination of ivermectin MDA for vector effect and antimalarials for parasite effect has been proposed in various scenarios.

### Efficacy.

The efficacy of ivermectin against malaria arises mainly from the mosquito-killing capacity of the drug when ingested during a blood meal. Additional sublethal effects that could affect the mosquito and/or the parasite in the mosquito and, therefore, malaria transmission, have also been described.^29,30^ Mosquitoes fed with ivermectin-containing blood have shown negative alterations on their fertility and flying ability.^29,30^ Likewise, the development of blood and liver-stage parasites has been inhibited by ivermectin in vitro and mouse models.^31,32^ However, the magnitude and mechanism of this effect in humans has yet to be fully studied. Because sublethal outcomes will require further elucidation, this report focuses exclusively on mosquito mortality as the primary determinant of ivermectin efficacy.

**Figure 1.**
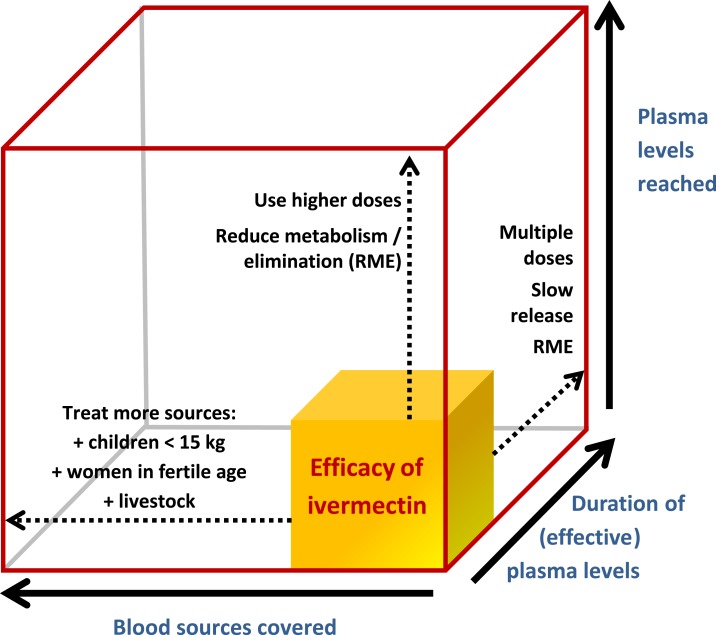
The theoretical efficacy of ivermectin mass drug administration based on three key parameters: (**A**) blood levels reached, **(B**) duration of blood levels, and **(C**) blood sources covered. This is a modified version of an original figure in by Chaccour and Rabinovich.^19^

The lethality of ivermectin is a function of three key parameters: 1) concentration: drug blood levels reached, 2) time: the duration of the drug circulating at effective concentrations in blood, and 3) coverage: the proportion of blood sources covered (Figure 1). In other words, the mosquito-killing effect is intrinsically related to the ivermectin concentration reached in the subject (human or animal), the time this concentration is sustained in the blood, and the number of subjects reached.

#### Concentration.

The common metric for ivermectin lethality is the lethal concentration 50 (LC_50_), which is defined as the concentration needed to kill 50% of the biting mosquitoes during a specific period of observation.^17^ Although the use of LC_50_ values are accepted by the scientific community, studies to assess the susceptibility of mosquitoes to ivermectin are not standardized, posing a challenge to deriving definite conclusions. For instance, LC_50_ data are often presented in a variety of time intervals, such as 24 hours, 5 days, 7 days, or 9 days, as shown in Table 2. Besides the disparity in time frame, the feeding methods for the LC_50_ assay also differ, potentially influencing the outcome (i.e., mosquitoes fed in vivo through a direct skin blood meal or in vitro via a membrane-feeding device). Moreover, in vitro experiments can be performed in two ways, using ivermectin-spiked blood or using blood from ivermectin-treated vertebrates. Last, blood meals may come from human or animal blood treated with ivermectin only, or in combination with antimalarials, adding extra variability.

**Table 2:** Susceptibility to ivermectin in a blood meal of key malaria vectors, ordered by species

Reference	Species	Method	Susceptibility
Gardner et al.^101^	*An. quadrimaculatus*	Feeding on treated dogs	24-hour-LC_50_: 6–12 ng/mL
Ouedraogo^102^	*An. gambiae*	Membrane: blood from treated humans in combination with artemether–lumefantrine	7-day-LC_50_: 8.6 ng/mL
Smit et al.^37^	*An. gambiae*	Membrane: blood from treated humans in combination with dihydroartemisinin–piperaquine	7-day-LC_50_: 3.4 ng/mL
Kobylinski et al.^103^	*An. gambiae* (G3 strain)	Membrane: in vitro mixture (human blood + ivermectin)	5-day-LC_50_: 22.4 ng/mL
Kobylinski^104^	*An. gambiae* (G3 strain)		7-day-LC_50_: 15.9 ng/mL
Fritz et al.^12^	*An. gambiae* s.l.	Membrane: in vitro mixture (cattle blood + ivermectin)	9-day-LC_50_: 19.8 ng/mL
Kobylinski et al.^105^	*An. dirus*	Membrane: in vitro mixture (human blood + ivermectin)	7 day-LC_50_: 55.6 ng/mL
	*An. minimus*		7 day-LC_50_: 16.3 ng/mL
	*An. campestris*		7 day-LC_50_: 26.4 ng/mL
	*An. sawadwongporni*		7 day-LC_50_: 27.1 ng/mL
Kobylinski [unpublished]	*An. dirus*	Membrane: blood from treated humans	10-day-LC_50_: 2.9 ng/mL
Kobylinski [unpublished]	*An. minimus*		10-day-LC_50_: 0.4 ng/mL
Sampaio et al.^106^	*An. aquasalis*	Membrane: in vitro mixture (blood + ivermectin)	5-day-LC_50_: 47.03 ng/mL
Kobylinski et al.^107^	*An. darlingi*	Membrane: in vitro mixture (blood + ivermectin)	7 day-LC_50_: 43.2 ng/mL
Chaccour et al.^22^	*An. arabiensis*	Feeding on treated cattle	10-day-LC_50_: 3.7 ng/mL
Fritz^23^	*An. arabiensis* (Dongola strain)	Membrane: in vitro mixture (cattle blood + ivermectin)	9-day-LC_50_: 7.9 ng/mL
Pasay^108^	*An. farauti*	Feeding on treated pigs	12-day-LC_99_: 2.4 ng/mL*

*An. = Anopheles.* In vitro and in vivo data for humans and/or animals are presented. Note the variability in LC_50_ values when using ivermectin-spiked blood or blood from treated vertebrates. In all cases, in vivo data produce a stronger lethal effect when calculating LC_50_s. LC50 not available. Results by Dreyer et al.^109^ showing an in vitro LC_50_ of 1,468 ng/mL for *An. albimanus* have been published, so they are included here for completeness but there are new, unpublished field data by the same team showing LC_50_ of 34 ng/mL or lower.

* Only LC99 available.

Equally challenging is the great variation of the LC_50_ between *Anopheles* species (Table 2)*.* In general, the mosquito vulnerability in a specific region will be partly defined by the least susceptible species being targeted (provided said species has a relevant role in transmission), which is considered as the dose-defining species and can vary somewhat across geographical areas. The main implication of this finding is that characterizing the major vectors in a geographic area and validating susceptibility test results from colony mosquitoes with those of wild-type mosquitoes will be a prerequisite to determine the dose-defining species and to implement ivermectin for vector control. Note that the time from feeding to mosquito death is dose-dependent (i.e., dependent on the ivermectin concentration in the blood at the time of biting) with higher doses shortening survival to a few hours, but even mosquitoes exposed to concentrations below 1 ng/mL still experienced reduced 28-day survival,^33^ which can contribute to reduce transmission. The total mosquito mortality achieved in the system with any given dose/regimen will depend on the area under the curve of the drug’s PK and the population coverage achieved.

#### Time.

Modeling predicts that the main driver on transmission reduction with ivermectin MDA is the duration of the drug concentration above mosquito-killing levels in the blood.^28^ The longer the ivermectin is available in the blood of treated subjects, the greater the impact on mosquito survival or fitness. Thus, the short-lasting presence of ivermectin represents an important challenge to be overcome through innovative approaches to enhance the duration of effect of ivermectin or through alternative active ingredients to be developed.

##### Dosing and regimen considerations.

The dose and regimen for ivermectin delivery must be designed to optimize impact while ensuring maximum safety. These are key factors that impact on the concentration and duration of ivermectin in blood and, therefore, the efficacy of the intervention. Ideally, mosquito-lethal concentrations of ivermectin would be sustained in the blood for as long as possible, while avoiding human toxicity and minimizing the number of MDA campaigns required. Ivermectin’s toxicity in humans is the result of cross-binding to GABA-gated channels which are only present in the central nervous system (CNS) and, hence, protected by the blood brain barrier (BBB). So, ivermectin toxicity in mammals is related to its level in the CNS, which is not necessarily related to the blood levels but to the integrity/maturity of the BBB and the activity of BBB-related efflux pumps such as the P-glycoprotein.^34^

The ivermectin label has been modified extensively over 30 years of treatment and prevention of NTDs. The current Federal Drug Administration (FDA)–approved ivermectin dose for onchocerciasis MDA is a single dose of 150–200 μg/kg yearly, although the possibility of quarterly use in individual patients is also included on the label for areas with high onchocerciasis transmission.^35^ For moderate to severe crusted scabies, three doses of 200 μg/kg within 2 weeks are recommended in the Stromectrol^®^ Australian label.^26^ Of relevance, the 400 μg/kg single dose yearly MDA is included on the Mectizan^®^ and the Stromectrol labels, both approved by the European Medicines Agency (EMA).^36^

**Figure 2.**
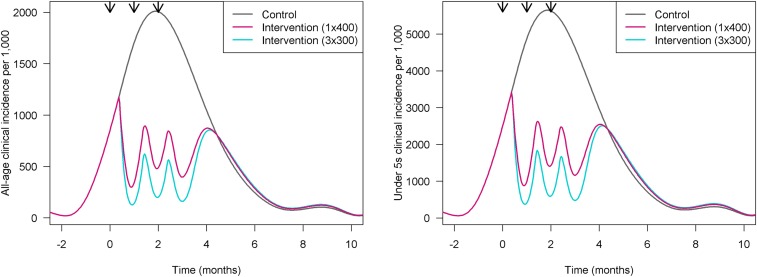
Modeled impact of the intervention across one transmission season in northern Mozambique. On the left, general population; on the right, children younger than five years (analysis by Hannah Slater).

Currently, two potential regimens are being considered for malaria clinical trials (see section Assessment of expected impact). One is the single 400 μg/kg dose that is in the EMA-approved ivermectin label, repeated three times during the malaria season. The second is a three-dose regimen of 300 μg/kg taken on 3 consecutive days (days 1–3), in combination with the artemisinin-based combination therapy (ACT), dihydroartemisinin–piperaquine, in three rounds administered during the malaria season.^37^ During the roadmap development, a modeling exercise was performed to evaluate the impact of both options in different transmission scenarios (Slater, unpublished). Figure 2 displays an example of a three-round deployment of ivermectin 1 month apart, each right at the start of the rainy season, at either the 1 × 400 or the 3 × 300 dose in a perennial transmission setting in northern Mozambique. The effect of the intervention is shown as the variation of clinical incidence of malaria over time for both all-age and under-five populations. The model shows that three doses of 300 μg/kg, used monthly for 3 months, could reduce clinical infection incidence by 50%, whereas a single dose of 400 μg/kg would reduce it by about 40%. Notably, although the difference between the two regimens is potentially measurable at the population level, other factors to be taken into account are the delivery logistics, cost-effectiveness, and community acceptability of each option. In the 3 × 300 regimen, the first dose is directly observed, whereas the remaining two doses are unobserved. Although this model was successfully used in SMC programs,^38^ it could lead to adherence concerns, particularly over time. There is a risk that the concurrent use of a single dose for NTDs and multiple-dose regimens for malaria may create confusion and affect acceptability by the community. Alternatively, the successful delivery of a single higher dose of 400 μg/kg may achieve greater effectiveness at the community level without a significant trade-off in efficacy. A thorough comparison of the two approaches is shown in Table 3.

**Table 3:** Advantages and disadvantages of the two ivermectin regimens for malaria being tested in clinical trials through 2023

	Advantages	Disadvantages
3 × 300 μg/kg daily doses per month “3 × 300”	Longer effect and, therefore, higher efficacy	Lower coverage expected (as evidenced in malaria community has experience with SMC and MDA 3-dose regimens)
		May have longer regulatory pathway, requiring additional safety and pediatric data for approval of a new dose and regimen
1 × 400 μg/kg dose per month “1 × 400”	Expected increased uptake and scalability with a single dose	Shorter duration of effect and, therefore, potentially lower impact
	Dose already approved under European Medicines Agency, in France and the Netherlands for LF MDA, may facilitate the regulatory process	Dose is used only in a few countries with LF
	Simpler to consider under national program guidelines and potential for synergies with NTD programs	
	Similarity to NTD programs could enhance community acceptance.	

LF = lymphatic filariasis; MDA = mass drug administration; NTD = neglected tropical disease.

Ivermectin dose is currently administered to humans during MDA campaigns using height as a proxy for weight with a validated dosing pole. Regardless of the final dosage for vector control, the development of a single dose for adults would facilitate operations and, most importantly, allow for easier co-administration, co-packaging, or even co-formulation with companion drugs for either NTDs or for malaria indication. In addition, height to weight ratios may need to be revalidated for higher doses.^39^

Because the efficacy of ivermectin is limited by the short-lived effect of the drug, long-lasting formulations of ivermectin or novel compounds with longer duration would represent an important improvement. Already, slow release formulations of ivermectin are currently under study showing positive preliminary data on efficacy and safety of implants,^22,40,41^ but scalability of implants could be challenging. There are other formulations such as patches or innovative gastric retention devices,^27^ which require further product development for safety as well as efficacy. Alternatively, a new class of molecules from the veterinary market known as isoxazolines has shown a better pharmacokinetic profile. When tested against malaria vectors, the predicted insecticidal activity of isoxazolines was 50–90 days.^42^ However, these molecules have received a U.S. FDA veterinary safety warning about potential for neurologic adverse events in dogs, and thus, further development for administration to humans for malaria impact is uncertain.^43^

#### Coverage.

The third driver of efficacy is coverage, which is the proportion of eligible blood sources treated. In areas where mosquitoes feed on both humans and animals, and livestock are targeted, coverage of livestock enters as a variable of coverage.^44^ In humans, the determinants of coverage are acceptance of the intervention, drug safety (through the scope of target population), and adherence. As in other public health interventions, outside of a clinical trial, good acceptance will require evidence of impact, aligned with strong community mobilization and engagement. In animals because it is a licensed product with benefits to health and productivity of herds, determinants of coverage as veterinary MDA for malaria include access, supply, and delivery chain at a timing to parallel human administration and the malaria season.

Ivermectin provides personal benefits against NTDs and ectoparasites, but it represents a novel paradigm of vector control for malaria, in which the benefit to the community is indirect, rather than directly to the individuals. Therefore, the concept of community impact needs to be well understood by the community and by the global and national policy-makers.

The fact that ivermectin has proven to be a safe drug for almost 30 years facilitates the pathway toward its repurposing for malaria use. However, higher or more frequent doses might be required for this new function, and thus, its safety profile may need to be re-evaluated to facilitate WHO evaluation. Last, acceptability and coverage might be negatively affected by low adherence if multiple-dose regimens are used. The shortest course of treatment could simultaneously ensure significant decrease in malaria transmission as well as programmatic feasibility and compliance.

#### Research and development agenda.

1. Definition of minimum mosquito data and stratification criteria for ivermectin implementation at scale.
2. Once efficacy is determined, validation of dosing pole for older children to simplify delivery. Dose-ranging studies and development of a single pill for adults that does not require weight adjustment would simplify operations.
3. Cross-study analysis of the impact in the field of 3 × 300 versus 1 × 400 of ongoing trials. Trade-offs between efficacy, programmatic feasibility and coverage, and community acceptance would be useful to guide efficient phase four studies of scale-up.
4. Development of improved formulations of ivermectin or other endectocides with acceptable safety profile and prolonged mosquito-killing time (e.g., slow release, patches, and new AIs).

#### Proven epidemiological impact.

As of May 2019, the only field clinical trial that demonstrated the impact of ivermectin MDA on malaria transmission was RIMDAMAL.^45^ This cluster-randomized trial evaluated efficacy against clinical malaria incidence in Burkina Faso after six ivermectin MDA delivered as single doses (200 μg/kg) every 3 weeks. A 20% reduction in malaria incidence in children ≤ 5 years old was shown with a community coverage of approximately 70%. The statistical significance of these findings has been the subject of debate.^46,47^ There are five ongoing or proposed trials with the two leading drug doses and regimens, in three rounds of drug administration (see section Ongoing/planned trials). Moreover, there are a number of other ivermectin trials of varying dose and regimen for NTD applications (see MESA Track https://mesamalaria.org/mesa-track).

### Safety.

#### Safety of different dosage and regimens.

Ivermectin has been used for more than 30 years with an excellent safety profile. More than 2.7 billion doses have been distributed both as individual treatment and as control of NTDs at the approved dose of 150–200 μg/kg, yearly, and with no major safety concerns.^48^ These programs are implemented in areas nonendemic for *Loa loa*, use a validated dosing pole to deliver to children > 15 kg, with a defined dose/kg, and do not administer the drug to pregnant women.

However, to attain the desired mosquito-killing effect for malaria applications, higher doses/more frequent regimens will be required. Given that the benefit of the community-based delivery of ivermectin for malaria is indirect, both the safety profile of the repurposed regimen and the risk-benefit analysis will be key for the success of this intervention.

Two dosing regimens are currently planned for evaluation in different trials. The safety of the proposed 400 μg/kg single-dose (1 × 400) scheme has been well established in clinical studies, as more than 60,000 thousand independent drug exposures have occurred in clinical trials (Supplemental Annex 1). No reported serious drug-related adverse events and only minor adverse events related to immune reactions from parasite death and clearance were observed (e.g., itchiness, myalgia, headache), with these disappearing within 1 week.

The safety profile of the second scheme proposed, a single daily dose of 300 μg/kg for 3 days (3 × 300), has been initially established in a single trial,^37^ and thus, it will require comprehensive safety assessment in larger clinical trials regardless of target disease. The key pharmacokinetic profiles of the proposed malaria regimens and those of other currently approved regimens are shown in Table 4.

**Table 4:** Main pharmacokinetic parameters for selected dosage schemes

	400 μg/kg single dose	300 μg/kg on days 1–3	Onchocerciasis, 150–200 μg/kg single dose	Moderate to severe scabies, 200 μg/kg three doses within 2 weeks
*C*_max_	63.8 [44–88.5]	69.4 [34.1–196.3]	38 [35–41]	38.3 [27.8–52.1]
AUC	2,353 [1,313–4,169]	5,000 [1,600–8,300]	1,032 [874–1,210]	3,532 [1970–6,266]
*T*_max_	5.3 [3.9–7]	48 + 3.9 48 + [0.75–7.6]	5.6	29 [27.8–30.3]

AUC = area under the curve. PK model by Hammann. All parameters in median [range] *C*_max_: ng/mL, AUC: ng·h/mL, *T*_max_: hours.

#### Exclusion criteria and drug interactions.

Existing exclusion criteria for administration of ivermectin against NTDs include co-infection with *L. loa* (> 30,000 mf/mL), pregnant women, children under 15 kg (or 90 cm as proxy), and women nursing babies that are younger than 1 week. This is because there is preclinical evidence of maternal and/or fetal toxicity at very high doses (10 to 150-fold) of ivermectin in pregnant mammals. A few clinical studies have evaluated the effects of inadvertent treatment during pregnancy without observed negative effects on either the mother or the newborn.^49–54^ A systematic review of the safety of ivermectin in inadvertently exposed mothers is in press (P. Nicolas, personal communication), and this can be supplemented with similar cases from active trials, but there is yet no standardized database of pregnancy exposures.

Although preclinical data in young monkeys show no adverse effects, there is limited data to support ivermectin use in younger age groups (under 15 kg of weight).^55^ Additional dose-ranging studies in young children would be needed to help develop clear guidance for either the use for therapy or prevention. Last, low ivermectin levels have been found in breast milk after treatment of a healthy mother with ivermectin. Given concerns about the maturity of the BBB in newborns under 7 days of age, nursing mothers in the first week after giving birth are currently excluded in MDA campaigns.

**Figure 3.**
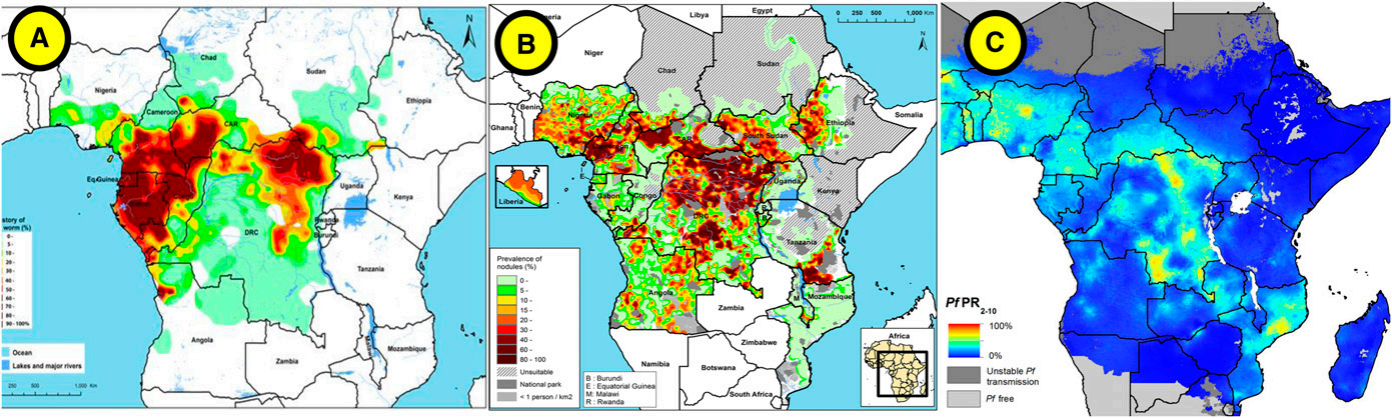
Overlap between selected *Loa loa*, onchocerciasis, and malaria-endemic areas in Africa. (**A**) Estimated prevalence of *L. loa* eye worm, (**B**) estimated prevalence of palpable *Onchocerca* nodules in the 20 African Programme for Onchocerciasis Control countries in 2011, and **(C**) *Plasmodium falciparum* parasite rate in 2–10-years-old children in 2015.

Areas known to be *L. loa* endemic have also historically been excluded from ivermectin MDAs in onchocerciasis/LF programs (e.g., areas of Angola, Cameroon, Chad, Ethiopia, and Gabon among others), although screening methods are beginning to be deployed. In patients with *L. loa* infection*,* the administration of ivermectin can result in fatal encephalopathy if the individual has an extremely high parasite density (> 30,000 mf/mL). Figure 3 shows areas with overlapping endemicity for *L. loa*, onchocerciasis/LF programs, and malaria. New diagnostic tools for real-time screening of *L. loa* with a test-and-not-treat strategy are potential advancements to expand MDA to areas traditionally excluded because of this risk.^56^

Additional studies on potential drug–drug interactions in the context of ivermectin MDA for malaria vector control may need to be assessed as several antiretrovirals and tuberculosis (TB) drugs share the same metabolic pathway (p450 CYP) with ivermectin,^57^ which could lead to 1) unexpected higher levels of ivermectin or other commonly used drugs, such as antiretrovirals or anti-TB drugs, potentially causing toxicity or 2) lower levels of ivermectin, antiretrovirals, or anti-TB drugs, thus reducing efficacy. Until safety is proven, field trials might want to consider using co-medication with these drugs as an exclusion criterion.

#### Research and development agenda.

1. Independent safety reviews of the 1 × 400 database and 3 × 300 regimen (ongoing).
2. Development plan for safety assessment under controlled conditions before field trials or regulatory and policy review of the 3 × 300 regimen.
3. Safety review in children under 15 kg and pregnant women are ongoing. Modeling indicates that acceptable coverage in the current eligible population will be reachable even with exclusion of these populations, and none of the ongoing/planned malaria trials include these populations. If needed for either treatment or prevention for any indication, additional studies on the safety for use of ivermectin in children < 15 kg and/or pregnant women would be required.
4. Current programs for MDA of ivermectin exclude visibly pregnant women without pregnancy testing. Additional data may be needed to support this approach early in pregnancy.
5. Creation of a pregnancy registry across all trials regardless of dose or indication; with enhanced input of the NTD community and an acceptable registry host.
6. Studies of drug–drug interactions between ivermectin and other relevant drugs such as antimalarials, antiretrovirals, and TB drugs.

### Ethical considerations.

As opposed to campaigns for NTDs, in which ivermectin provides a direct benefit to individuals by reducing the parasite burden, MDA for malaria is an intervention with an indirect community benefit and requires careful attention to key ethical concepts in public health. Such ethical considerations will be relevant both at the individual level during clinical trials (i.e., study participants), and at a broader level as the intervention advances to scale-up (i.e., country leaders, policy makers, civil society, academia, and community leaders).

Although early data from limited studies suggest a direct effect of ivermectin on *Plasmodium* liver stages in the host,^31^ other data contradict this finding, and therefore, the possibility of individual protection requires validation. If it exists, the antiparasitic activity seems to be minor, and is not yet well understood. Thus, this report exclusively focuses on the ivermectin indirect impact on human health at the population level as the primary malaria benefit of the intervention, without consideration of the possible direct impact on the parasite itself.

The idea of indirect benefit through a delayed community effect is not unique to malaria prevention interventions. For example, it has a precedent in the current efforts to develop a malaria transmission–blocking vaccine.^58^ This concept also echoes the “herd immunity” impact of live attenuated vaccines, where vaccination provides benefit to those who remain unvaccinated, and extends beyond prevention of one individual. Moreover, the community effect of an ivermectin MDA also has parallels with IRS, where all households can be protected when high coverage is achieved.

Ivermectin is a good candidate for an intervention with indirect benefits because of its overall safety profile previously established through its extensive use against NTDs. However, a risk-benefit analysis that considers the known risks of any adverse effects and the benefit of lower chances of malaria infection is a critical component of full policy analysis before implementation of this strategy. Discussions with community leaders and local health workers about the advantages and limitations of ivermectin MDA will be required to ensure good acceptability and compliance, starting before the clinical trials. From a communication perspective, it will be key to convey simultaneously the idea that ivermectin, although it is a pill, does not provide individual protection or treatment against malaria, and therefore, clearly communicate that it is a complement to vector control with ITNs and IRS. The fact that a drug is being delivered might lead to certain confusion because it can be interpreted as a treatment for malaria, which could result in lower compliance with national malaria prevention and control measurements (e.g., use of ITNs, care-seeking behavior). For that reason, evaluation of the effectiveness of that communication will be important. Ensuring that this is understood at the community level will be critical for both NTDs and malaria MDA programs, and will need effective, validated communication tools during trials and beyond.

During clinical trials, all participants receive a clearly written informed consent, with which they can accept or refuse to engage in the study (this is extended to minors through their guardians and even to livestock owners); all planned trials are being conducted under national ethical review, often with multiple layers of oversight. Importantly, the message that a high coverage is necessary to achieve community protective effect (i.e., greater than 80% of the eligible population, based on modeling studies^27,28^) must also not be perceived as coercive to any individual’s willingness to participate in the program. Upon the transition to scale-up, the national plan should aim for equity of access to the intervention under defined parameters for priority communities.

### One health.

Current ivermectin veterinary indications for horses, cattle, pigs, sheep, and small ruminant species include the treatment and control of gastrointestinal nematodes, lungworms, and ectoparasites. Oral, topical, and injectable formulations are available at a range of concentrations from multiple veterinary approved generic producers.

**Figure 4.**
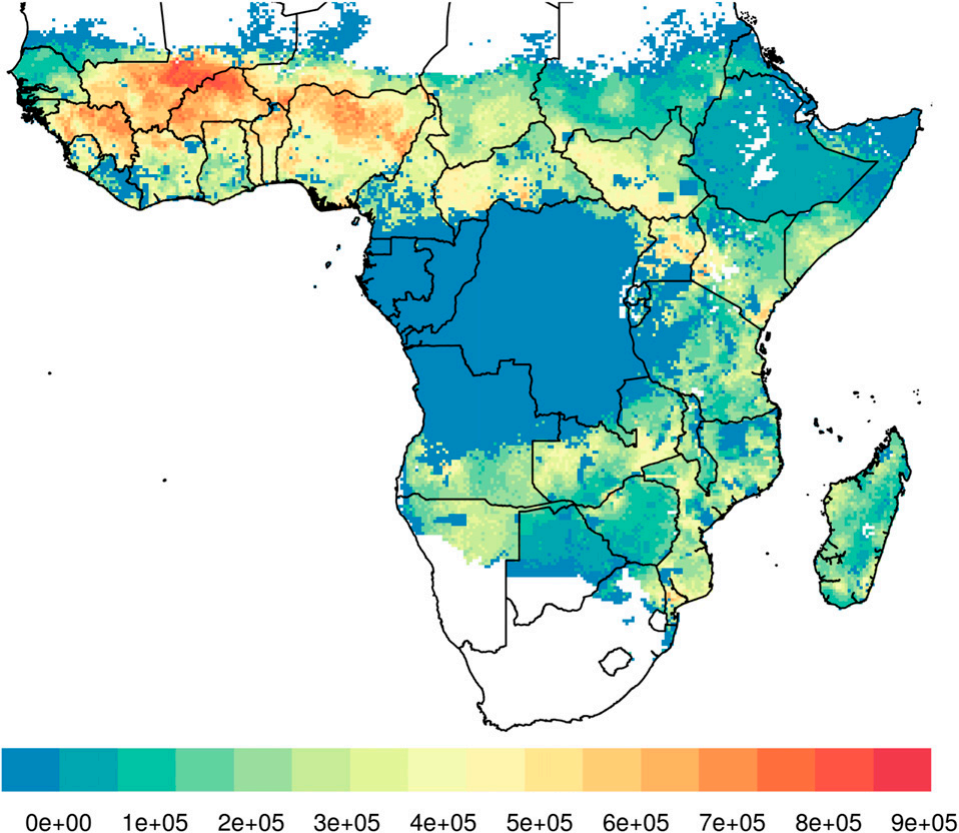
Areas where high cattle density coincides with high malaria prevalence in 2–10-year-old children (Inbahale et al).^24^

In many rural African communities, herds are brought into areas contiguous to human residence each evening, and their blood sustains the *Anopheles* mosquito population, hence creating the opportunity for effective intervention with ivermectin. A dual strategy providing ivermectin MDA to both humans and livestock will be particularly valuable in areas where both malaria prevalence and cattle density are high, as described by Imbahale et al.^24^ (Figure 4). A coordinated ivermectin MDA to humans and livestock could target zoophagic vectors that evade human-centered approaches and, thereby, contribute to reducing the residual transmission of malaria. Moreover, ivermectin treatment will also directly benefit herds and their owners by reducing the burden of other parasites, increasing productivity and, thereby, improving overall living conditions of the communities.

A single veterinary ivermectin injection can last up to 6 weeks and is typically used in high-income countries to treat entire herds, but less frequently implemented in low-income settings. Pharmacokinetic data show that ivermectin levels in cattle capable of killing > 95% of *Anopheles arabiensis* in 10 days can be sustained for 6 weeks after a single injection of 600 μg/kg with a 3.15% formulation (Ivomec gold^®^ [Boehringer Ingelheim Animal Health Argentina], which has a longer meat withdrawal period of up to 120 days, preventing the use for meat for 3 months).^59^ This is important as the time frame in which mosquitoes become infective after feeding on an infected person ranges from 10 to 16 days (hence, it is particularly important to reduce the proportion of mosquitoes that reach this age). In pigs, a single dose of 200 μg/kg with a 1% formulation of ivermectin can result in 1.5 weeks of mosquito-killing effect.^40,60^

Some of the factors determining the efficacy of livestock-delivered ivermectin will be the livestock species and presence (e.g., surface area of the animal, variation in ivermectin metabolism by species, proximity of the animals to humans, and the livestock/human ratio), the local mosquito species composition, and the local mosquito feeding preference (e.g., the degree of zoophagy versus anthropophagy) as determined by blood meal analyses.

All of these determinants require careful assessment with human epidemiological endpoints in clinical trials before deployment of ivermectin in livestock. In addition, the environmental impact of the intervention and its programmatic feasibility in Africa should be established in such trials. Additional risks such as alterations in mosquito behavior must be investigated. For instance, a modeling exercise suggests that in cases of two or more vector species competing for the same niche, intensive veterinary use of ivermectin could result in one species shifting to more anthropophagic behavior (Dighe and Slater, unpublished).

However, as a common veterinary drug, the safety of ivermectin in a number of animal species has been widely tested. Exclusion criteria for livestock use would include milking mothers and animals planned for slaughter within a defined window according to established withdrawal periods for safe human consumption of animal products (see section The regulatory process for veterinary use).^61^

Ivermectin is the first-in-class endectocide currently being experimentally evaluated in livestock as a proof of concept to determine if this strategy might control malaria transmission.^12,22,23,62^ Other endectocides can be deployed in livestock, including eprinomectin, which has the remarkable advantage of having a zero milk/slaughter withdrawal period.

Although use of other endectocides in livestock may ultimately be preferable to reduce selective pressure on mosquitoes or reduce milk withdrawal times, ivermectin can be considered for proof of concept trials. Along these lines, rotating or using livestock endectocides in mosaics could help prevent resistance in mosquitoes or the primary target of these drugs, gastrointestinal parasites in livestock. Ivermectin MDA for malaria prevention to livestock poses, however, a series of similar challenges in the areas of regulation, supply and delivery, environmental exposure, as well as policy and financing, which will need to be carefully assessed to determine the cost/benefit of campaigns targeting both humans and livestock (see section Regulatory pathway and policy recommendation pathway).

#### Research and development agenda.

1. Mapping of main livestock species to be targeted, including veterinary/husbandry practices, where trials to evaluate veterinary impact are planned
2. Scalable system for mapping livestock and relevant mosquito species where veterinary use is considered
3. Evaluation of impact of extensive MDA to livestock in evolutionary selection of mosquitoes
4. Impact of veterinary MDA on resistance to primary helminth targets, drawing from experience where this has been already used extensively.
5. Potential use of endectocides without milk withdrawal times such as eprinomectin.
6. Indirect risks and benefits of veterinary MDA, including collateral production benefits
7. Environmental impact of veterinary MDA in the tropical context.

### Ongoing/planned trials.

As of October 2019, there are at least six clinical trials preparing to evaluate ivermectin MDA against malaria either ongoing or under development. The studies vary in design and will use several approaches for community delivery of ivermectin, including synchronization with other MDAs, combination with the administration of antimalarial drugs, and inclusion of treatment of livestock (Table 5). Each trial will have distinct primary outcomes and will provide insights on different aspects of ivermectin MDA.

**Table 5:** Trials using ivermectin to reduce malaria transmission through 2023 ordered by the time to first results

Trial name	Lead researcher	Country	Dose	Drug combination	First results
MASSIVE	Umberto D’Alessandro	The Gambia	3 × 300	DHA-P MDA	2020
RIMDAMAL II	Brian Foy	Burkina Faso	3 × 300	Seasonal malaria chemoprevention	2020
TBC	Kobylinski and Sattabongkot	Thailand	1 × 400	Ivermectin alone	2020
TBC	Karine Moiline	Burkina Faso	N/A	Ivermectin to livestock	2020
TBC	Anna Last	Guinea-Bissau	3 × 300	DHA-P MDA	2021
BOHEMIA	Rabinovich and Chaccour	Tanzania and Mozambique	1 × 400	Ivermectin alone + ivermectin to livestock	2021 and 2022

DHA-P = dihydroartemisinin–piperaquine; MDA = mass drug administration; N/A = not-applicable.

Although these studies are independent and individually funded, the investigators are collaborating on critical design and dose selection issues. Sharing information on the selection of primary and secondary outcomes of the trials and having a common denominator to assess coverage of MDA (e.g., census versus no census) would facilitate comparability of results among studies and understanding the effect of variable coverage on efficacy. Similarly, the methods for monitoring resistance should be comparable across individual trials. Creation of a database that incorporates data from all the trials, including safety reviews and a registry for unplanned pregnancy exposure, would be an important asset and could help accelerate the policy and regulatory process. Last, cooperation between researchers to coordinate how to present and discuss the results to audiences, such as relevant country stakeholders, the WHO, regional organizations, regulatory authorities, would greatly facilitate further policy and funding mechanisms for the implementation of ivermectin MDA, assuming positive trial results and a WHO supportive policy. In addition, it will be important that the various communities and organizations, including NTDs, malaria, non-governmental organizations (NGOs), academia, and government, to name a few, work through the learnings in implementation at each site and the translation agenda to scale across geographies.

### Assessment of expected impact.

The potential health and economic impact of ivermectin MDA in humans (cases and costs averted) has been estimated using projections under different scenarios (Chaccour et al., unpublished). This analysis assumes that the results from the funded BOHEMIA clinical trials (i.e., BOHEMIA, see section Assessment of expected impact) will be positive and followed by a WHO policy recommendation on the use of ivermectin MDA for malaria vector control. The model has been developed for the period of 2023–2027 with assumptions that clinical trials will produce supporting evidence by 2022 that the results and intervention will be acceptable to the community and that additional funds are available for translation and implementation at scale in a number of African countries. The projection of impact of a 400 μg/kg single dose was made under the following conditions:

1. A group of 20 African countries were identified as potential first implementers based on the burden of malaria, lack of drug contraindications, and current political stability. The selected countries were classified as early (2023) or late (2025) implementers, based on their previous experience with ivermectin programs for other indications.
2. Uptake was conservatively defined as piloting districts encompassing at least 5% of the country’s population at risk with a 3% increase yearly (conservative estimate of uptake based on the SMC program). In these districts, the target coverage of the intervention was 80% of the eligible population (i.e., only including non-pregnant women and children above 15 kg) or 64% of the general population.
3. The potential efficacy of the ivermectin MDA regimen was defined as 20% reduction in clinical incidence (minimally required criterion in WHO’s PPC) or 40% (projected estimate based on modeling).
4. Ivermectin is expected to have important synergies with current core vector control tools (i.e., ITNs and IRS) by reducing mosquito biomass. A very conservative 5% cumulative reduction in cases was factored in as current models cannot predict synergies (i.e., 5% of the predicted cases in any year do not occur in the next year).
5. The reduction in malaria cases was adjusted to four potential global scenarios based on the work of Griffin et al.^63^ for the GTS (WHO GTS).^5^
6. Financial cost savings were calculated using the final figure of cases averted and the average cost of non-complicated and complicated malaria cases in Africa,^64^ applied to either 1 or 3% of cases as “complicated.”
7. Intervention costs were calculated using preliminary final dosage form (FDF) cost and the delivery costs based on published figures for ivermectin delivered as a single dose for NTDs.
8. Final savings and costs per case averted were calculated with the above data.

**Figure 5.**
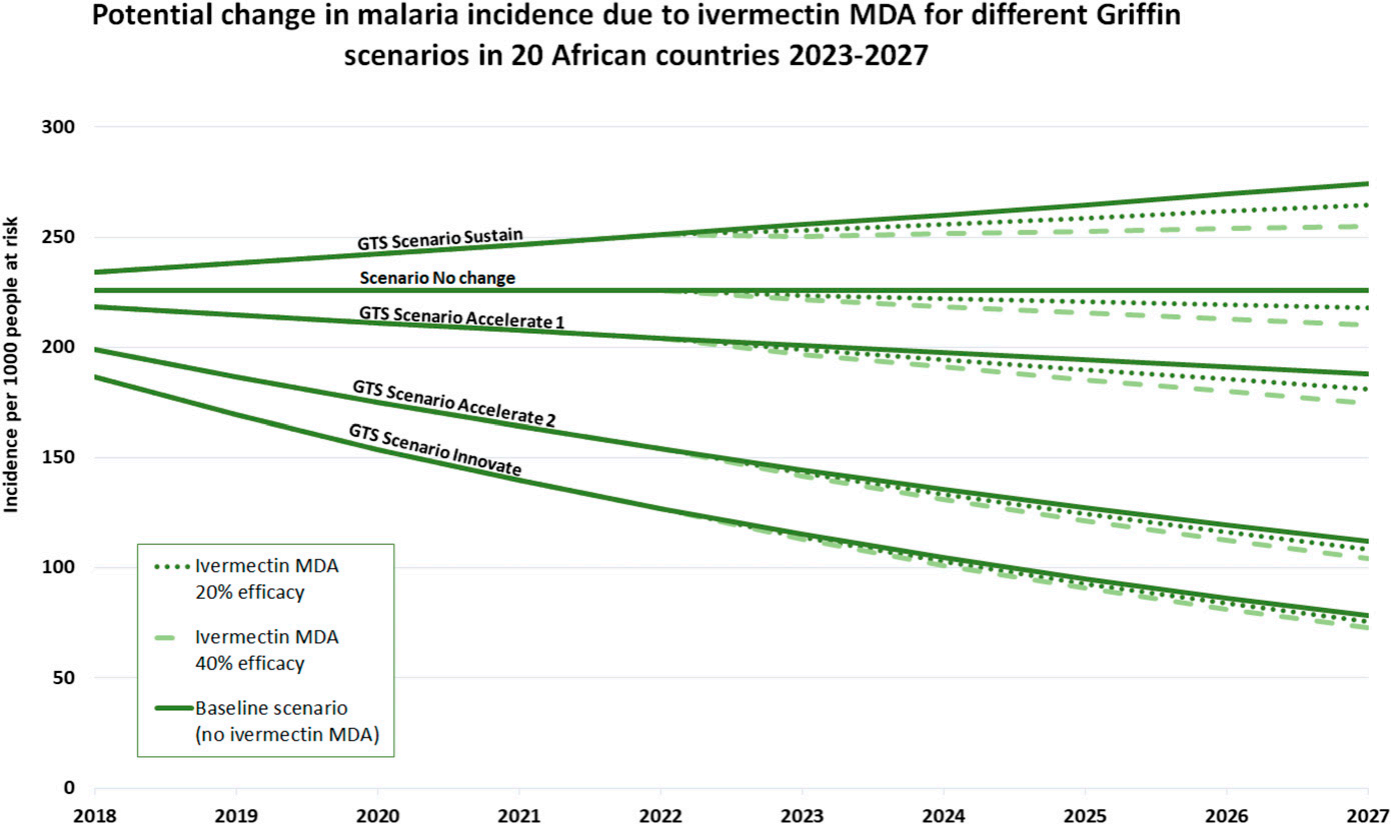
Malaria incidence per 1,000 population at risk in 20 selected countries during the 2018–2027 period and calculated additional impact attributable to the BOHEMIA intervention from 2023–2027.

Figure 5 shows the predicted changes in clinical malaria incidence under the four Griffin scenarios: Sustain, Accelerate 1, Accelerate 2, and Innovate for the selected 20 early introduction African countries. The dotted lines show the additional impact attributable to an ivermectin MDA, considering the minimum required efficacy of 20%, as well as the alternative estimated efficacy of 40%. Taking these results into account, further calculations predicted that the intervention could avert between 11,000 and 65,000 deaths, and between 5.2 million and 32 million cases from 2023 to 2027, resulting in cumulative averted financial costs between US$32 million and US$208 million.

### Resistance.

Broad community deployment of ivermectin to reduce malaria transmission has the potential to enhance the impact on diseases already treated with this drug, such as onchocerciasis, LF, and scabies, but it also runs the risk of generating resistance in some NTDs, as well as in mosquitoes and veterinary parasites. Therefore, any approach to using ivermectin MDA against malaria should take into account that the current (2019) and evolving primary indications of the drug must be protected while designing strategies that minimize the ability for mosquitoes to develop resistance.

#### Resistance to current indications.

There are valid concerns about increasing selective pressure on filariae with wider use of ivermectin, but this risk is not well documented. Reports of ivermectin-resistant *Onchocerca* have been the subject of debate.^65–68^ These reports refer to suboptimal response against *Onchocerca* with persistent microfilaremia and early repopulation. The balance of evidence suggests that presence of suboptimal response does not threaten the control of onchocerciasis as a public health problem. Moreover, as part of the risk mitigation strategy for filarial resistance, triple therapy with albendazole–diethylcarbamazine (DEC)–ivermectin has shown dramatic impact at killing the worm after 2 years of treatment and will likely become the new approach for LF eradication.

Ivermectin is also under evaluation for MDA against soil-transmitted helminths (STHs) because adding ivermectin will increase the impact of deworming programs by targeting *Strongyloides*, which is not affected by albendazole-based programs. The risk of resistance in STH has been described as higher than for filariae, although risk mitigation strategies are also being considered. Combination therapy of ivermectin and another anthelmintic to increase the effect on partially affected parasites, such as *Trichuris,* could lower the risk of resistance.^69^ Furthermore, the concerted application of water, sanitation, and hygiene programs along with antihelminthics in STH areas are functional resistance risk mitigation strategies because impact is generated via an alternative route.

The dose and regimens proposed for malaria are higher and/or more frequent than that indicated for some NTDs (higher than filariae, lower than those under consideration for STHs), which could have a larger impact on the overall biomass of filariae circulating on certain areas, effectively reducing the number of parasites exposed to the drug in the longer term and making the window of selection for resistance narrower.

The primary purpose of veterinary ivermectin (and most other endectocides) is to enhance agricultural production by improving livestock health. The efficacy of ivermectin against endo- and ectoparasites of veterinary importance should be monitored in the context of a more intensive, albeit intermittent, use for malaria.

#### Mosquito resistance.

Note that mosquitoes have sporadically received low concentrations of ivermectin for decades at a massive scale through MDAs against NTDs. No obvious evidence of resistance to ivermectin has been seen to date, although limited testing hampers the ability to draw conclusions from this absence of evidence. However, the potential emergence of resistance to ivermectin in malaria vectors needs to be carefully monitored and risk mitigation strategies created before the implementation of ivermectin MDA.

To date, ivermectin drug-class resistance in arthropods of human and veterinary importance has been associated with a wide range of mechanisms: reduced cuticular penetration,^70^ mutation of the target, glutamate-gated chloride ion channel,^71^ accumulation of GABA,^72^ and metabolic resistance due to overexpression of xenobiotic pumps^73,74^ and cytochrome P450 isoenzymes.^74,75^

The available data on ivermectin cross-resistance with pyrethroids is conflicting. In some studies, permethrin-resistant houseflies, cockroaches, and head lice were susceptible to abamectin or ivermectin.^76–79^ In other studies, permethrin-resistant cockroaches, houseflies, and *Aedes aegypti* mosquitoes were less susceptible to abamectin or ivermectin.^80^ In addition, ivermectin tolerance mediated by transcription of P450s and xenobiotic pumps can be induced in ticks, head lice, and *Drosophila melanogaster.*^81–83^ Overall, this suggests that differential mechanisms of ivermectin tolerance and resistance occur and that cross-resistance is also possible via at least one of these mechanisms.

Based on observational studies, ivermectin MDA has proven effective even in areas with high levels of pyrethroid resistance among *Anopheles*,^84^ but increased selective pressure from repeated dosing may change this. Thus, urgent attention should be placed on characterization of how *Anopheles* could develop tolerance and resistance to ivermectin. This research could identify molecular markers for surveillance that would indicate population susceptibility or rising resistance to ivermectin. However, molecular markers are only associative, and so phenotypic studies (mortality assays with wild-type *Anopheles)* should also be periodically conducted, particularly in areas with evidence of ITN effectiveness decay.

In the context of livestock treatment, ivermectin has been shown to be able to stay active in the whole water system up to 127 days.^85^ Mass cattle treatment could expose mosquito larvae in their aquatic habitat via cattle defecation. This could expose both zoophagic and anthropophagic *Anopheles,* as their larval habitat during the rainy season are formed from accumulation of rainwater in animal hoof prints, small puddles, ponds, and temporary animal watering holes, all of which are often contaminated with livestock excreta. The longer residency time of endectocide in injected cattle also increases the window in which adult mosquitoes may imbibe sublethal concentrations, with the potential increase in resistance as susceptible populations are slowly reduced by the drug.

Until molecular markers of resistance are identified and developed into a validated assay, susceptibility testing in mosquitoes will have to rely on one or more common phenotypic assays, such as feeding ivermectin in a blood meal or sugar water to adult mosquitoes. This would require well-characterized standards of serum (frozen or lyophilized) or sugar solution that could be shipped to all sites and applied in a standard way to local F1 or F2 generation mosquitoes. The amount of work to overcome local variability in mosquito rearing, willingness to blood feed through a membrane, etc., is not trivial, but developing common approaches and standard operating procedures (SOPs) for resistance monitoring strategies could provide critical early warnings and make it feasible to more easily generate comparable data at multiple sites.

Theoretically, a rudimentary approach to identifying resistance markers is to force resistance in laboratory colonies of one or more *Anopheles* species. The approach is to give blood meals with increasing, sublethal doses of ivermectin and metabolites to each generation until resistance is observed. Establishment of ivermectin-resistant *Anopheles* colonies is complicated by the fact that sublethal ivermectin concentrations can still inhibit fecundity. If successful, genomic tools could then be used to identify resistance markers by comparison of parental and selected mosquito strains albeit with the known limitations in external validity of resistant colonies generated in the insectary.

Ideally, it would be useful to incorporate a common strategy to monitor ivermectin susceptibility at the baseline and end of field MDA trials to determine resistance development over the course of time. Simple approaches for monitoring include using sugar meals with ivermectin to detect potential changes in the LC_50_. If phenotypic changes are found from tests before and after interventions and compared with untreated areas, these samples could be molecularly tested to try and understand the genetic basis for resistance.

#### Resistance risk mitigation strategies.

The risk of resistance emergence in mosquitoes after veterinary application needs to be acknowledged, discussed, and monitored. Several risk mitigation strategies have been proposed. The creation of refugia^86^ (untreated populations) could be a useful approach to manage resistance risk for veterinary parasites, which de facto would be implemented by the proposed selection criteria for treatment (exclusion of milk and/or soon-to-market livestock as per current regulations). An alternative strategy discussed would be to implement ivermectin in mosaics within herds or by using a different endectocide class in an area where humans receive ivermectin. Refugia would similarly be created in implementation of human ivermectin MDA, given the indicated exclusion of young children and pregnant women from MDA campaigns.

To prevent mosquito resistance, early research into optimal combination with other vector control approaches should be performed; in addition, attention to other potential uses of ivermectin, both for NTDs as well as malaria, that could increase the exposure and hasten the appearance of resistance, such as ivermectin-based sugar baits^87,88^ or wall linings,^89^ should be discussed and evaluated as part of risk management.

In summary, ivermectin MDA in humans and relevant veterinary species is proposed as a complementary vector control strategy, and all trials to evaluate its impact on malaria are occurring in the context of ITNs, with or without IRS. As a result, ivermectin would be part of combinatorial strategies with multiple routes to their mosquito targets (via direct cuticular contact, blood meal, or sugar ingestion) and with complementary modes of action that should all limit resistance development among both intestinal parasites and vectors. It follows that each component in a combination strategy should have differential modes of action to help preserve the others. Given that ivermectin is the only safe and registered endectocide available to humans at this time and for the foreseeable future, and that it is designated an essential medicine by the WHO as an NTD tool, efforts should be put in place to preserve its NTD action and decrease the risk of potential resistance in human parasites and mosquitoes.

##### Research agenda.

1. Develop the right tools to monitor resistance in other target organisms such as filariae and STH by the NTD community
2. Investigate whether ivermectin has important cross-resistance with other vector control tools (i.e., resistance to pyrethroids)
3. Generate a standard ivermectin susceptibility assay for mosquitoes
4. Continue active monitoring of vector resistance status throughout malaria MDA ivermectin campaigns

### Environmental impact.

The environmental impact of intense ivermectin use in livestock has been extensively documented in places of large-scale use against helminths in Europe and the United States. However, there are limited data of the use in tropical regions in the context of African small-holder herding practices. In addition, there are no data on the environmental impact of mass administration of ivermectin to humans. There are reasons to posit that this would not be higher than in temperate regions because of the effect of higher temperature and humidity that would hasten drug degradation. On the other hand, the environmental impact is dependent on the relative susceptibility of flora and fauna in specific tropical versus temperate areas, which are presently unknown. Overall, environmental safety and impact are important steps in the evaluation process of vector control tools.

Following veterinary administration, ivermectin and its metabolites are excreted in feces and may enter into different environmental compartments (e.g., dung, soil, surface water, or groundwater). Dose and route of administration of ivermectin, as well as diet, affect the levels of ivermectin and its metabolites in the feces, whereas environmental factors such as climate, population density, soil type, vegetation, and waste management practices influence how long these compounds persist in the environment in addition to where and how they accumulate.^85^ Ivermectin can persist in the dung of treated livestock for weeks to months, potentially affecting nontarget insects, many of which are dung-dwelling species considered essential for dung degradation. Ivermectin and its metabolites can also accumulate in soil and in water because it is not readily biodegradable in aquatic systems. Although this drug is not toxic to mammals, microorganisms, or plants, it can be toxic to aquatic invertebrates (i.e., *Daphnia magna*), algae, and fish.^87^

Finally, the NTD experience shows that the pharmaceutical industry gives great importance to the appropriate disposal (via incineration) of expired medicines. Plans to do so need to be embedded in the malaria MDA trials and, later, would need to be built into implementation plans.

## REGULATORY PATHWAY AND POLICY RECOMMENDATION PATHWAY

Ivermectin has been licensed since the 1980s to treat a number of parasitic helminths in both humans and animals. Its use at massive scale for NTD MDA programs has been under a donation model with product approved under WHO-defined stringent regulatory authorities. Although ivermectin is listed on the WHO Essential Medicines list and WHO has had an open invitation to manufacturers for submission of ivermectin for prequalification (which is specific to the manufacturer, even for a generic product), there are currently no prequalified ivermectin producers. This is likely due to the donation model for onchocerciasis and LF, the current primary applications at large scale (see section Manufacturing and procurement), whereas generic manufacturers do provide ivermectin for treatment under national regulatory approval in various countries. Thus, repurposing ivermectin for malaria vector control and other pending applications will require, beyond a WHO policy recommendation for its use for malaria control, WHO prequalification of any product that is not approved by a stringent regulatory agency as defined by WHO, to allow for national procurement with funds from multilateral or bilateral donors. Prequalification, defined further in the following paragraph, can be given in the context of a range of indications and, thus, serves as a pathway to approval for a new indication.

### Regulatory pathway for use as a malaria vector control intervention for human malaria.

**Figure 6.**
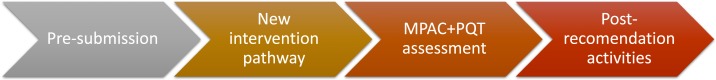
Steps in the WHO evaluation system for new vector control tools.

A policy recommendation for ivermectin by WHO will require a thorough review across several departments of the agency, including the disease-specific programs good manufacturing practices (GMPs) and NTD and their respective advisory committees (i.e., MPAC, vector control advisory group [VCAG], as well as the prequalification team [PQT], and essential medicines, among others). The evaluation system for new vector control tools, which was revised in 2017, can be divided in four general phases: pre-submission, new intervention pathway, parallel GMP/NTD department assessment (through MPAC for malaria/STAG for NTDs) and PQT inspection, and post-recommendation activities (Figure 6, Table 6).^90,91^

**Table 6:** Summary of the WHO prequalification process for new vector control tools^91^

Phase	Primary outcome	Steps	Factors	Pathway designation
Pre-submission	Define evaluation pathway	Pre-submission to PCC	Pre-submission coordination committee	Pathway designation
New intervention pathway	Validated public health value of product class	Concept review and data definition	VCAG	Defined data required to validate public health value and support a WHO policy recommendation
		Development of assessment standards	VCAG product developer	Developed efficacy test guidelines, SOPs, quality and safety standards, and criteria
		Manufacturer-led data generation	VCAG product developer	Clinical trial results
		PQT inspection	PQT product developer	Report from manufacturing facility inspection
		Data assessment and recommendation to MPAC	VCAG	Final VCAG report to MPAC
Good Manufacturing Practices/Neglected Tropical Disease/PQT Assessment	1) Policy recommendation issued 2) Product prequalified	MPAC/STAG assessment	MPAC	MPAC meeting report
		PQT assessment	PQT	Product listing
Post-recommendation activities	Programmatic use	Country health authority review	MoHs NMCP	Country policy issued
		Country regulatory review	MoHs Reg. authorities	Product registered in country
		Country procurement	MoHs GFTAM/UN/PMI	Product procured by countries
		Country use	MoH NMCP	Roll out and monitoring
Post-PQT activities	Ongoing inspections and assessments	Post-PQT activities	PQT	

MPAC = Malaria Policy Advisory Committee; PCC = preferred product characteristics; PQT = prequalification team; VCAG = Vector Control Advisory Group.

#### Phase 1: Pre-submission

The first step of the assessment is the pre-submission of the proposed clinical trial and/or draft product label to a single-entry portal managed by PQT. The feedback from the PQT will determine the evaluation pathway that a potential vector control tool must follow 1) Prequalification Pathway (i.e., if the product is part of a class with an existing WHO policy recommendation), or 2) New Intervention Pathway (i.e., if the product does not belong to a class with an existing WHO policy recommendation). Accordingly, the consideration of a new indication for malaria control will fall under the New Intervention Pathway,^91^ thus requiring the need to evaluate its health value as well as other parameters, including mosquito impact and overall safety.

To allow for VCAG’s review of the evidence generated, manufacturers and research groups currently working on the subject are invited to take protocols specifically to VCAG for detailed review before generating the evidence.

#### Phase 2: New Intervention Pathway

The New Intervention Pathway is managed by the GMP and NTD, with a single-entry pathway for VCAG and others to perform the assessment of the product dossier.^92^ Advice will be given to the applicant in terms of data required, trial design, risk assessments, and product specifications. In addition, VCAG will provide feedback on assessment standards and requirements (i.e., SOPs, quality and safety standards, etc.) through periodic interaction with the applicant. Because ivermectin is a drug, VCAG review will likely include other relevant expertise, including drug prequalification for drug safety and participation of NTD and GMP staff.

Vector Control Advisory Group’s guidelines state that a requisite for a policy recommendation is to show an epidemiological proof in at least two randomized controlled trials conducted in two different settings with data collected across two consecutive transmission seasons.^93,94^ Additional recommendations by VCAG for the design of research studies, based on the input to other products reviewed, may include the following:

1. Early development/standardization of means of testing for resistance (both in vectors and in endoparasites)
2. Data on residual efficacy in feces and other environmental safety issues (nontarget organisms)
3. Risk assessment
4. Data on vector population recovery times and the duration of effect needed to sustain/achieve long-term population reduction
4. Strong community engagement strategies
5. Product pricing, deployment plans, and costing are not a requisite, but cost-effectiveness assessment is relevant to inform formulation of a policy recommendation
6. Stringent regulatory approval could facilitate product regulatory approval in endemic countries, but policy recommendation from WHO is on the critical path for the malaria indication as well as policy recommendation in countries

#### Phase 3: MPAC assessment/policy recommendation

After a positive recommendation on the value of the product from VCAG as a vector control tool, it will be further evaluated by the corresponding policy advisory committee for malaria (MPAC). In parallel, following a manufacturer request for prequalification, PQT conducts a formal review and/or inspection of the manufacturing facilities to ensure compliance with quality standards. This process should include an evaluation of the programmatic suitability and of the operational research agenda.

Given satisfactory results of both processes, WHO will then issue a policy recommendation and the product will become “first in class.” Operational guidelines for implementation will be released in parallel to the policy. However, prequalification of the product will be based on the assessment and, although the policy refers to a generic product, the prequalification approval will be linked to specific manufacturer’s facility evaluation for ‘GMP’ as well as other technical evaluations of product. Because this is the first drug to go through VCAG, the process may be tailored to generics.

#### Phase 4: Post-recommendation/post-prequalification activities.

This phase refers to the plan and work needed at the country level for issuing national policies based on WHO’s recommendation, as well as the requirements for product registration, procurement, and implementation at scale. In addition, continuous inspection of product quality and manufacturing facilities will be performed by the PQT.

#### Action Items

1. Engage with VCAG as an entry point for guidance before the trials to ensure that methods and data comply with their requirements
2. Enhance communication across clinical trial groups to ensure comparable key endpoints across trials to provide VCAG and regulatory bodies with a robust body of evidence
3. Develop a pathway assessment for country trial to policy and implementation steps following country evidence of ivermectin impact

### Regulatory process for veterinary use.

For malaria, mass use of endectocides in livestock has a dual pathway for evaluation and approval. Whereas global health authorities review the efficacy endpoint (i.e., reduction of malaria transmission) and product quality, animal health authorities are responsible for the regulatory approval of the intervention for veterinary use (i.e., a specific dose, regimen, or formulation) and for its overall effect on animal health. In addition, the use of drugs in animals raised for human consumption is assessed by the international Joint FAO/WHO Expert Committee on Food Additives and the Codex Alimentarius Commission. These bodies define the acceptable daily intake of the drug and the appropriate withdrawal times for milk and meat consumption according to the dose/regimen selected and to the livestock species being treated (Table 7).

**Table 7:** Withdrawal times for slaughter (WDI) or milking (WDT) in animals treated with ivermectin * FDA approved withdrawal times

Livestock species route of administration	Ivermectin dose (mg/kg body weight)	Meat WDI (days)	Milk WDT (days)
Cattle			
Subcutaneous 1%	0.2	35*	47
Subcutaneous 3.15%	0.6	120–140	N/S
Oral	0.2	24*	28
Topical	0.5	48*	53
Swine			
Subcutaneous	0.3	18*	NA
Oral	0.1	5*	NA
Sheep			
Oral	0.2	11*	NA
Goats			
Subcutaneous	0.2	35	40
Oral	0.2–0.4	14	9
Topical	0.5	NA	7

NA = Not available. Other values based on Food Animal Residue Avoidance Databank–recommended withdrawal intervals.

In the case of ivermectin MDA in livestock, the proposed dose and formulation are already approved, and the product is available. This class of products is licensed under stringent regulatory authorities, and there are licensed veterinary formulations already approved in many malaria-endemic countries. The pathway to policy recommendation for veterinary use for malaria may require review of the WHO malaria recommendations and engagement with the WHO health community by the FAO, but this will probably adapt to the unique requirements of ivermectin as an existing drug with multiple applications for veterinary endpoints.

## TRANSITION AND IMPLEMENTATION AT SCALE

If the effectiveness of ivermectin MDA against malaria is proven and achieves a WHO policy recommendation, additional work will be needed to facilitate country introduction and implementation at scale. The transition to scale-up will include activities such as the inclusion of the concept of intervening in residual transmission in the national strategic plans; evaluation and development of sustainable delivery models appropriate to country context; determination of appropriate entomological and epidemiological monitoring and evaluation strategies; engagement with key stakeholders at global, regional, and national level; and engagement with generics manufacturers as suppliers in the context of a high-volume, low-price market. Modeled on the introduction of antimalarials for community prevention of malaria (i.e., SMC and MDA), it is assumed that the initial roll out will begin in a subset of districts or provinces of a group of early introducer countries. Presumably, the earliest implementers would be those countries experiencing significant levels of residual transmission in which clinical trials were conducted.

Thus, the WHO policy recommendation is critical, but important work to translate the recommendation as a new intervention to country-led programs should be planned for in parallel to evidence creation, particularly as data emerges on efficacy in the various trials. Ideally, timely guidance from WHO on what additional information is needed is an important part of the process. For example, consideration is needed as to how ivermectin would be optimally incorporated within the package of existing interventions.

Given potential programmatic synergies, collaboration with the well-established NTD programs will be key to national implementation plans to enhance impact as well as avoid any competition for sharable resources (e.g., staff, transportation, etc.). However, key aspects such as drug regimens (i.e., single dose annually for onchocerciasis and LF versus multiple times a year for malaria), timing of the intervention (i.e., malaria control would be distributed during the rainy season, whereas NTD is not limited and moreover often optimally delivered in the dry season, when transport is easiest), and supply chains (i.e., procured versus donated) need to be carefully considered in the creation of country plans. Subnational mapping of priority target areas may be different for both indications.

In any case, the distinction between ivermectin to treat NTDs and to control the malaria vector needs to be clearly conveyed to the targeted communities. Data and learnings from the various clinical trials on how to best communicate that ivermectin MDA is a drug to kill the mosquito, but is not an antimalarial, should be an additional point of collaboration in the clinical trial phase that can be applied to scale-up post-policy recommendation.

Last, financing of ivermectin MDA by multilateral agencies such as the Global Fund or bilaterals such as PMI will likely require a recommendation from WHO, an adoption by national malaria strategic plans, and either first tier-approved or prequalified product. Thus, part of the work required for implementation at scale will focus on the accomplishments of these key milestones (see section 5.5 for financing).

### Manufacturing and procurement.

Current onchocerciasis and LF programs are based on the donation of ivermectin by the pharmaceutical company Merck to the Mectizan Donation Program. The corporation has recently announced a new commitment to include ivermectin in triple therapy for LF through 2025, which will increase the demand for the donation program over the next decade. For the donation program, prequalification is not required because Mectizan is a stringent regulatory approved product (e.g., by the FDA and EMA). Although the donation program has provided enormous health benefits, the model has limited the investment incentives for other manufacturers to supply the drug for limited therapeutic indications. As a consequence, although ivermectin is on the WHO Essential Medicines list, no product has yet been prequalified (as of October 2019).

A policy recommendation for ivermectin MDA against malaria would help build a high-volume/low-price generics business case for manufacturers because it will result in the potential need for procurement by malaria donors and/or country governments of billions of additional ivermectin tablets over the next decade. This would create a business investment case for generic manufacturers to seek WHO prequalification to support countries to request international funds for the procurement of the drug. In addition, the potential inclusion of other new indications such as the treatment or MDA to control scabies^95^ (recently included as an NTD by the WHO^96^) and ivermectin MDA with other drugs for STHs^95^ would bolster the generics business case. Given the constant evolution of the NTD landscape and emerging uses of ivermectin, the global supply needs for the drug will require careful tracking and anticipation to meet future and realistic demand projections.

The good news is that as an established generic product, production of ivermectin is streamlined as the drug is manufactured at ambient temperature, with available film-coating processes, and is technically scalable, as has already been demonstrated by the donating manufacturer. As a generic drug, scalable production protocols and testing procedures are both publicly available (e.g., a bioequivalence study protocol has been recently published by WHO). In addition, there are several manufacturers of active pharmaceutical ingredients (APIs) with self-reported productions above 50 tons per year.^18^ Most of the global manufacturing of APIs is of veterinary grade, whereas the process for human APIs requires additional quality controls and documentation. As guidance, only around 2.5 tons of APIs per year are needed to treat 180 million people with the current single-dose onchocerciasis regimen, and thus, the pathway to increased supply can be managed if the increase in demand is communicated, planned, and phased.

There are a number of generic companies in Europe, Asia, Latin America, United States, and Africa that produce other drugs under GMP, with WHO and/or SRA (e.g., FDA and EMA) reviewed and approved facilities. Given the potential interest by generics manufacturers in new markets at high volume/low price, the timeline to go from positive trial results to availability, in the context of already GMP/qualified active ingredient, would be relatively short.

Technically, it would also be feasible to manufacture 18–20 mg tablets as single dose for adults, and this would make dosing more effective and potentially cheaper at scale. Additional work to demonstrate the potential value, the impact on supply chain management and procurement, as well as to build the investment case for this tablet size would likely be required. Although a pediatric formulation may be important in the long term, it will require further development and it is not a current priority because children < 15 kg do not receive this drug for malaria MDA or any other control campaigns. However, pediatric interest could increase if scabies or STH MDAs are approved and implemented at scale because it would broaden the eligible population.

Finally, in terms of procurement and management of the drug, the current label states that storage should be under 30°C up to 3 years; however, it is handled with fewer restrictions at final delivery by existing programs. There may be value in evaluating the supporting data, as well as the stability and programmatic suitability of the specific formulations used.

### Interactions with NTDs and other national programs.

Assuming the ongoing trials provide evidence that ivermectin MDA for malaria has a robust effect, it will be critical to begin collaborative approaches with the national NTD programs to develop models for delivery. The lessons learned from 30 years of ivermectin MDA deployment should guide the complex operational aspects of scaling-up the use of ivermectin under the new indication. Moreover, insights from other malaria MDA trials and programs, including SMC and ACT MDA, should be also considered. Emerging MDA programs, such as azithromycin to reduce overall child mortality,^97^ and other national campaigns such as immunization, may also need to be considered for synergy and operational alignment.

Given the extensive geographical overlap of malaria- and NTD-endemic areas (Figure 3), there are a number of potential health and operational benefits as well as challenges for a national ivermectin platform targeting multiple diseases, and both will need to be actively discussed by program leaders. For instance, ivermectin NTD programs could benefit from an enhanced impact due to the delivery of a second annual dose of the drug, a strategy that has been recommended but still not well implemented. In addition, where malaria extends to wider areas than NTDs, the malaria program could create buffer zones around areas targeted for onchocerciasis/LF elimination, preventing the reintroduction of infections. This strategy has proven successful in West Africa as part of the Onchocerciasis Control Program.^98^

Among the challenges of dual ivermectin programs is the current misalignment of the dose and regimen, as well as product source (i.e., one donated and the other purchased), for drug distribution. The ivermectin dosage proposed for malaria is higher than the one for onchocerciasis control, although the 400 x 1 dose is within the range recommended for LF. Moreover, the regimen under evaluation for malaria is not only more frequent but also time sensitive to the mosquito proliferation around the rainy season. However, an approach recommended to address suboptimal response (i.e., rapid return of microfilaridermia after treatment) in *Onchocerca volvulus* infections is to increase the frequency of ivermectin delivery from annually to every six or 3 months,^99^ although this is not broadly implemented given isolated existence of suboptimal response. Ideally, the higher ivermectin dose for malaria should be sufficient to cover both vector control and NTD strategies. Nevertheless, additional work would be needed to understand and plan for co-administration of other drugs such as DEC or albendazole with ivermectin to satisfy other disease requirements.

Prior experience with community deployment for NTDs originally started using costly mobile teams, and then drug delivery was transferred to village health workers and, through a greater involvement of the communities (i.e., community-directed), with village volunteers minimally supervised by government personnel.^100^ However, this annual volunteer approach may not be appropriate for three sequential, monthly ivermectin campaigns for malaria. Moreover, in places where both malaria and NTD programs co-exist, transparent roll out of two different sources of ivermectin and an agreed on mechanism for distribution and communication strategies will be needed. Preliminary engagement of leaders at global and national levels indicates strong support for consideration of complementary strategies.

### Stakeholder engagement and community uptake.

Engagement with different stakeholders will be critical from the planning of clinical trials through to the establishment of sustainable delivery programs. The range of relevant stakeholders will vary according to the phase of the effort and to established country partners in the malaria, NTD, Maternal and Child Health, and veterinary programs. “Stakeholder” is a broad umbrella that includes local and national government, academic, and civil society leaders; in-country NGOs; journalists; and potentially NGOs of relevance, such as RTI, Sightsavers, MSF, CHAI, and others. Similarly, funders can include bilaterals (e.g., PMI and DFID), multilaterals (The Global Fund, UNICEF, and other UN agencies), and national funds. Specifically, designing the national malaria strategies and the post-policy roll out of the intervention will represent crucial moments where stakeholder engagement will be instrumental for country ownership, as well as for leveraging and strengthening the capacities at the institutional and community levels.

Given the uniqueness of the intervention, in which a known drug is used to reduce malaria transmission through an indirect, community effect, the acceptability by community members will drastically affect uptake and coverage. Collaboration between in-country institutions with previous outreach experience and community leaders will be important. As early as at clinical trials stage, formative research will help understand the factors influencing the acceptability of ivermectin for malaria as well as how to effectively convey the vector control nature of the intervention.

### Funding.

Besides the health impact of the intervention, cost-effectiveness analyses will be an important driver for countries and funding agencies to support the use of ivermectin for malaria control. Clinical trials should be designed to provide economic data for this analysis, although complementary modeling exercises can help estimate the cost of implementing ivermectin MDA in real settings, including delivering the drug to humans only and to humans and livestock.

The key opportunity is to examine the results from different clinical trials as they emerge to approach funding bodies in advance and facilitate program launch after a WHO policy recommendation, as well as to assure ivermectin supply. For instance, a WHO recommendation is a prerequisite for submitting proposals to the GFATM, but time to approval will depend on when in the cycle of funding a country request is made and whether countries include residual transmission plans in their strategic plans. Therefore, parallel briefings to national malaria control programs (NMCPs) on projected study design and timelines to emerging data, for consideration of inclusion of general language related to interventions to address residual transmission in national strategic planning as early as possible, will be particularly useful for early introduction countries. This intervention may also be relevant to other potential sources of funding, including national funds, World Bank loans or grants, implementing NGOs, and bilateral agencies supporting malaria programs. Briefings as data are generated and the impact case is refined will facilitate translation to scale once a policy recommendation is available. In addition, an adaptive mapping of the timings for regulatory approval, policy recommendation, ivermectin demand creation, and final costs of implementation, among other aspects, will be key to inform manufacturers and funding bodies.

An analysis of different funding bodies and specific requirements for each of them would facilitate the process. This is particularly true for potential funding of veterinary products because careful analysis and presentation will need to clearly present the value proposition for malaria programs, and the source of funding is not clear at this time.

#### Research agenda.

1. Projections of ivermectin demand and supply taking into account other NTD indications as well as malaria control
2. Creation of a rigorous business case for ivermectin manufacturing and country supply, including demand projections to facilitate industry engagement and supply of prequalified ivermectin at a generic pricing structure
3. Reassess ivermectin shelf life (currently labeled as 3 years) and whether additional heat stability studies are needed for storage above 30°C degrees
4. Assess the potential for single dose indication and formulations
5. Potential for alignment of malaria and NTD control programs, as well as other national health campaigns
6. Analysis of most cost-effective integrated delivery system options in collaboration with other programs
7. Development of effective communication tools regarding the vector control nature of the intervention
8. Evaluation of the health and economic impact of intervention in the longer term

## CONCLUSION

**Figure 7.**
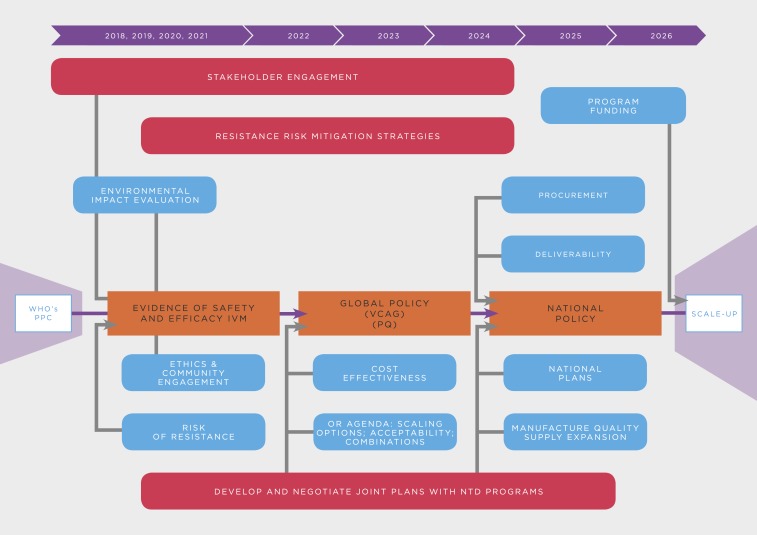
General overview of the key milestones, as well as the factors involved in the potential inclusion of ivermectin into the malaria toolbox.

The Roadmap illustrates the pathway to assess the potential repurposing of ivermectin as a complementary vector control tool for malaria and its subsequent large-scale implementation. Given the complexity of the overall process, the different actions and steps required from proof of concept to field deployment have been carefully described here. As a summary, Figure 7 provides a visual representation of the key milestones to be achieved and the factors involved in the inclusion of ivermectin into the malaria toolbox.

## Supplemental Annex

Supplemental materials
