# Supplementary material for: A Roadmap for the Development of Ivermectin as a Complementary Malaria Vector Control Tool
Source: Am J Trop Med Hyg. 2020 Feb 6;102(2 Suppl):3–24. doi: 10.4269/ajtmh.19-0620 (PMC7008306; doi:10.4269/ajtmh.19-0620)
Supplement: Supplementary file 1 [file tpmd190620.SD1.docx]

**Annex 1.** Summary of independent exposures to 400 mcg/kg dose

| **Reference** | **Highest single dose** | **Max. freq.** | **Max. number of doses** | **Maximum total dose**  **(period)** | **Population** | **Sample by dose**  **(only ivermectin)** | **Sample by dose  (ivermectin combined with other drugs)** | **Adverse events** | **Notes** |
| --- | --- | --- | --- | --- | --- | --- | --- | --- | --- |
| Dembele et al 2010  [1] | 400 mcg/kg | 6-monthly | 4 | 1,600 mcg/kg (2 years) | 51 adults infected with Wuchereria bancrofti microfilaria (LF) | N/A | 150 mcg/kg + albendazole 400 mg: 26 patients  (104 exposures to ivermectin 150 mcg/kg)  400 mcg/kg + albendazole 800 mg: 25 patients  (100 exposures to ivermectin 400 mcg/kg) |  |  |
| Chosidow et al 2010  [2] | 400 mcg/kg | Days 1 and 8 | 2 | 800 mcg/kg | 812 patients at least 2 years of age and weighted 15 kg with live lice not eradicated by topical insecticide used 2 to 6 weeks before enrollment | 400 mcg/kg: 398 patients | N/A | There were no significant differences in the frequencies of adverse events between the two treatment groups (ivermectin vs. 0.5% malation lotion). | 812 patients randomly assigned to either ivermectin or malathion |
| Ramaiah et al 2007  [3] | 400 mcg/kg | 12-15 months intervals (except the 9^th^ round that was given 2 years after the 8^th^ due to drug procurement constraints) | 9 | 3,000 mcg/kg (in 9-10 years) | Patients at risk of LF who weighted more than 15 kg, were not pregnant, and were healthy | 400 mcg/kg: 8,526 patients  (76,734 exposures to ivermectin 400 mcg/kg) | N/A |  | Other study arm: diethylcarbamazine (DEC)  Control group (placebo) was discontinued after two rounds |
| Ismail et al 2001  [4] | 400 mcg/kg | Single | 1 | 400 mcg/kg  (once) | 47 male asymptomatic microfilaraemic subjects with Wuchereria bancrofti infection | N/A | Ivermectin 200 mcg/kg + albendazole 400 mg: 16 patients  Ivermectin 400 mcg/kg + albendazole 600 mg: 15 patients | All 3 treatments were clinically safe and well tolerated. | Third study arm: albendazole 400 mg with diethylcarbamazine citrate (DEC) 6 mg/kg |
| Cooper et al 1999  [5] | 600 mcg/kg | Single | 1 | 600 mcg/kg  (once) | 40 healthy males with moderate to heavy *Onchocerca* infection | 150 mcg/kg: 18 patients  400 mcg/kg: 8 patients  600 mcg/kg: 8 patients | N/A | High-dose ivermectin was associated with more-severe reactions, more-profound eosinopenia, and higher circulating levels of interleukin-5 and eosinophil-derived neurotoxin, compared with the standard dose. | Fourth study arm: placebo |

| Ismail et al 1998  [6] | 400 mcg/kg | Single | 1 | 400 mcg/kg | 50 male asymptomatic microfilaraemic subjects with Wuchereria bancrofti infection (LF) | N/A | ivermectin 400 mg/kg + albendazole 600 mg: 13 patients  ivermectin 400 mg/kg + diethylcarbamazine citrate (DEC) 6 mg/kg: 10 patients | Follow up time: 15 months  All 4 regimens were well tolerated and clinically safe, although mild, self-limited systemic reactions were observed in all treatment groups.  The results suggest that alb/iver is a safe and effective single dose regimen for suppression of microfilaraemia in bancroftian filariasis that could be considered for control programmes. | Other study arms: albendazole 600 mg; albendazole 600 mg + DEC 6 mg/kg |
| --- | --- | --- | --- | --- | --- | --- | --- | --- | --- |
| Dreyer et al 1998  [7] | 400 mcg/kg | Single | 1 | 400 mcg/kg | 31 males with Wuchereria bancrofti microfilaraemia | N/A | Ivermectin 200 mcg/kg + DEC 6 mg/kg: 11 patients  Ivermectin 400 mcg/kg + DEC 6 mg/kg: 10 patients  Ivermectin 200 mcg/kg + (5 days later) DEC 6 mg/kg: 10 patients |  |  |
| Bockaire et al 1998  [8] | 400 mcg/kg | Yearly | 2 | 800 mc/kg  (in 2 years) | Patients in communities at risk of microfilaraemia | N/A | Ivermectin 400 mcg/kg + DEC 6 mg/kg: 1,098 (approx.) patients  (2,154 exposures to ivermectin 400 mcg/kg) | No side-effects occurred that required medical  intervention other than antipyretics. | Other study arms: DEC 600 mg/kg |
| Addiss et al 1997  [9] | 400 mcg/kg  (200-400 mcg/kg; mean: 273 mcg/kg) | Single | 1 | 400 mcg/kg | Children aged 5-11 years in communities at risk of LF | 200-400 mcg/kg (mean 273 mcg/kg): 28 patients | Ivermectin 200-400 mcg/kg + albendazole 400 mg: 24 patients | Systemic adverse reactions did not differ significantly between children who received ivermectin alone and those who were treated with ivermectin and albendazole. | Other study arms: placebo; albendazole 400 mg |
| Nguyen et al 1996  [10] | 400 mcg/kg  (after 3 doses 6-monthly doses of 100 mcg/kg) | 6-monthly | 3  (plus 3 previous doses of 100 mcg/kg) | 1,200 mcg/kg  (in 1.5 years)  (1,500 mcg/kg in 3 years) | 872 (average of 3 MDAs) individuals aged 3 years or more in a community at risk of microfilaraemia | 100 mcg/kg: 872 patients  400 mcg/kg: 872 patients  (2,616 exposures to ivermectin 400 mcg/kg) | N/A | The 400 micrograms/kg dosage was well tolerated and compliance was excellent. |  |
| Moulia-Pelat et al 1996  [11] | 400 mcg/kg | yearly | 2 | 800 mcg/kg  (in 2 years) | 57 males infected with Wuchereria bancrofti microfilaraemia | 400 mcg/kg: 19 patients  (38 exposures to ivermectin 400 mcg/kg) | Ivermectin 400 mcg/kg + DEC 6 mg/kg: 19 patients  (38 exposures to ivermectin 400 mcg/kg) | After the first treatment, 34 patients had adverse reaction (22 of level 1 and 12 of level 2). After the second treatment, 10 patients had adverse reactions (7 of level 1 and 3 of level 2). None of the adverse reactions were serious (recovery after up to 48 hours).  The adverse reactions were not significantly associated with treatment group.  The adverse reactions were significantly correlated with the initial level of microfilaraemia. | Other study arm: DEC 6 mg/kg |
| Ismail et al 1996  [12] | 400 mcg/kg  (preceded by 20 mcg/kg 3 d before) | Fortnightly | 12 | 4,820 mcg/kg  (in 6 months) | 37 asymptomatic microfilaraemic subjects with Wuchereria bancrofti infection | 20 mcg/kg: 11 patients  400 mcg/kg: 14 patients  (168 exposures to ivermectin 400 mcg/kg) | N/A | Mild, self-limited systemic reactions to therapy were observed in all 3 treatment groups. Local reactions, such as development of scrotal nodules, were observed in several subjects in the DEC and ivermectin groups. | Other study arm: DEC 3 mg/kg + DEC 10 mc/kg |
| Dreyer et al 1996  [13] | 400 mcg/kg | Every 2 weeks | 12 | 4,800 mcg/kg (in 6 months) | 15 men who had living adult Wuchereria bancrofti detected in the scrotal area by ultrasound | 400 mcg/kg: 15 patients  (180 exposures to ivermectin 400 mcg/kg) | N/A | Systemic adverse reactions were observed in 6 patients. The most frequent reactions,  which were transient and occurred within 24 hours  following the first dose, included fever, headache,  haematuria, and myalgia. The severity of these  reactions was related to pretreatment mf density. |  |
| Dreyer et al 1995  [14] | 400 mcg/kg | Single | 1 | 400 mcg/kg | 15 men who were infected with W. bancrofti. | 400 mcg/kg: 15 patients |  |  |  |
| Dreyer et al 1995  [15] | 400 mcg/kg  (400 mcg/kg preceded 4 d earlier by either placebo or very small doses (20 mcg/kg) of ivermectin) | Twice (different doses) | 1 | 420 mcg/kg  (in 5 days) | Asymptomatic adults infected with bancroftian filiriasis | 200 mcg/kg: 11 patients  220 mcg/kg (20 mcg/kg + 200 mcg/kg (4 d later)): 11 patients  420 mcg/kg (20 mcg/kg + 400 mcg/kg (4 d later)): 11 patients | Ivermectin 20 mcg/kg + DEC 6 mg/kg: 12 patients | Adverse reactions were well tolerated with all regimens, the reactions being significantly more generalized (i.e., fever) following ivermectin and localized (i.e., scrotal inflammatory nodules around dying adult worms) following DEC. | Other study arm: DEC 6 mg/kg; DEC 1 mg/kg + DEC 6 mg/kg |
| Moulia-Pelat et al 1994  [16] | 400 mcg/kg | Single | 1 | 400 mcg/kg | 30 male Wuchereria bancrofti carriers | 100 mcg/kg: 5 patients  400 mcg/kg: 5 patients | Ivermectin 400 mcg/kg + DEC 1 mg/kg: 5 patients  Ivermectin 400 mcg/kg + DEC 3 mg/kg: 5 patients  Ivermectin 400 mcg/kg + DEC 6 mg/kg: 5 patients |  | Other study arm: 6 mg/kg |
| Moulia-Pelat et al 1994  [17] | 400 mcg/kg  (after 4 doses 6-montly of 100 mcg/kg) | Single | 1 | 800 mcg/kg  (in 3 years) | 43 male Wuchereria bancrofti carriers | 100 mcg/kg: 22 patients  400 mcg/kg: 21 patients | N/A | The proportion  of patients with side effects was not significantly different  between the 2 groups. |  |
| Martin-Prevel et al 1993  [18] | 400 mcg/kg | Single | 1 | 400 mcg/kg | 31 Loa loa-infected subjects with low-to-moderate parasitemia (7-7,700 microfilaria/ml) | 300 mcg/kg: 16 patients  400 mcg/kg: 15 patients | N/A | The clinical tolerance of treatment was very good, and except in one case, only mild adverse reactions were observed, with pruritus being the most common symptom. There were no significant changes in blood or urine function test results or in hematologic results, except for a pronounced eosinophil reaction. |  |
| Kazura et al 1993  [19] | 400 mcg/kg | Twice (different doses) | 2 | 420 mcg/kg (in 5 days) | 50 men with bancroftian filariasis | 220 mcg/kg: 10 patients  220 mcg/kg (20 mcg/kg + 200 mcg/kg (4 d later)): 10 patients  420 mcg/kg (20 mcg/kg + 400 mcg/kg (4 d later)): 10 patients | N/A | No significant side effects (e.g., acute adenolymphangitis, fever lasting more than eight hours, hypotension) were observed in any of the five treatment groups. | Other study arms: DEC 6 mg/kg (once); DEC 1 mg/kg + DEC 6 mg/kg (4 d later); |
| Addiss et al 1993  [20] | 400 mcg/kg  (plus low clearing doses of ivermectin (20 mcg/kg), DEC (1 mg/kg), or placebo 4 days before) | Twice (different doses) | 2 | 420 mcg/kg (in 5 days) | 59 persons with Wuchereria bancrofti microfilaremia | 220 mcg/kg (20 mcg/kg + 200 mcg/kg (4 d later)): 9 patients  220 mcg/kg (placebo + 220 mcg/kg (4 d later)): 10  420 mcg/kg (20 mcg/kg + 400 mcg/kg (4 d later)): 9 patients | 20 mcg/kg ivermectin + DEC 6 mg/kg: 11 patients | Adverse reactions, which were generally mild, occurred more frequently with ivermectin than with DEC. | Other study arm: DEC 6 mg/kg; DEC 1 mg/kg + DEC 6 mg/kg |
| Cartel et al 1992  [21] | 400 mcg/kg | Single | 1 | 400 mcg/kg | 17 females aged 21 to 84 years and 20 males aged 26 to 57 years Wuchereria bancrofti carriers | 400 mcg/kg: 37 patients | N/A | Adverse reactions were observed in 65% of female and in 70% of male carriers; they were of grade > or = 2 in 35% of carriers in both groups. None as considered serious; they all disappeared in 24-48 hours. The main symptoms were headache, fever > or = 37.5 degrees C and myalgia in females. One male vomited 3 hours after treatment; as a result, the drug was not ingested and no decrease of microfilaraemia was noted. Twelve days afterwards, he was given a second 400 mcg/kg dose, he experienced again a grade 1 reaction and his microfilaraemia fell to zero. |  |

**References**

1. Dembele B, Coulibaly YI, Dolo H, Konate S, Coulibaly SY, Sanogo D, et al. Use of high-dose, twice-yearly albendazole and ivermectin to suppress Wuchereria bancrofti microfilarial levels. Clin Infect Dis. 2010;51 11:1229-35. <https://www.ncbi.nlm.nih.gov/pmc/articles/PMC3106228/pdf/51-11-1229.pdf>.

2. Chosidow O, Giraudeau B, Cottrell J, Izri A, Hofmann R, Mann SG, et al. Oral ivermectin versus malathion lotion for difficult-to-treat head lice. N Engl J Med. 2010;362 10:896-905; doi: 10.1056/NEJMoa0905471.

3. Ramaiah KD, Das PK, Vanamail P, Pani SP. Impact of 10 years of diethylcarbamazine and ivermectin mass administration on infection and transmission of lymphatic filariasis. Transactions of the Royal Society of Tropical Medicine and Hygiene. 2007;101 6:555-63; doi: 10.1016/j.trstmh.2006.12.004. <https://academic.oup.com/trstmh/article-abstract/101/6/555/1890807?redirectedFrom=fulltext>.

4. Ismail MM, Jayakody RL, Weil GJ, Fernando D, De Silva MS, De Silva GA, et al. Long-term efficacy of single-dose combinations of albendazole, ivermectin and diethylcarbamazine for the treatment of bancroftian filariasis. Transactions of the Royal Society of Tropical Medicine and Hygiene. 2001;95 3:332-5.

5. Cooper PJ, Awadzi K, Ottesen EA, Remick D, Nutman TB. Eosinophil sequestration and activation are associated with the onset and severity of systemic adverse reactions following the treatment of onchocerciasis with ivermectin. The Journal of infectious diseases. 1999;179 3:738-42; doi: 10.1086/314647.

6. Ismail MM, Jayakody RL, Weil GJ, Nirmalan N, Jayasinghe KSA, Abeyewickrema W, et al. Efficacy of single dose combinations of albendazole, ivermectin and diethylcarbamazine for the treatment of bancroftian filariasis. Transactions of the Royal Society of Tropical Medicine and Hygiene. 1998;92 1:94-7; doi: 10.1016/S0035-9203(98)90972-5. <http://dx.doi.org/10.1016/S0035-9203(98)90972-5>.

7. Dreyer G, Addiss D, Santos A, Figueredo-Silva J, Noroes J. Direct assessment in vivo of the efficacy of combined single-dose ivermectin and diethylcarbamazine against adult Wuchereria bancrofti. Transactions of the Royal Society of Tropical Medicine and Hygiene. 1998;92 2:219-22.

8. Bockarie MJ, Alexander ND, Hyun P, Dimber Z, Bockarie F, Ibam E, et al. Randomised community-based trial of annual single-dose diethylcarbamazine with or without ivermectin against Wuchereria bancrofti infection in human beings and mosquitoes. Lancet (London, England). 1998;351 9097:162-8; doi: 10.1016/s0140-6736(97)07081-5. <https://www.thelancet.com/journals/lancet/article/PIIS0140-6736(97)07081-5/fulltext>.

9. Addiss DG, Beach MJ, Streit TG, Lutwick S, LeConte FH, Lafontant JG, et al. Randomised placebo-controlled comparison of ivermectin and albendazole alone and in combination for Wuchereria bancrofti microfilaraemia in Haitian children. Lancet (London, England). 1997;350 9076:480-4; doi: 10.1016/s0140-6736(97)02231-9. <https://www.thelancet.com/journals/lancet/article/PIIS0140-6736(97)02231-9/fulltext>.

10. Nguyen NL, Moulia-Pelat JP, Cartel JL. Control of bancroftian filariasis in an endemic area of Polynesia by ivermectin 400 micrograms/kg. Transactions of the Royal Society of Tropical Medicine and Hygiene. 1996;90 6:689-91.

11. Moulia-Pelat JP, Nguyen LN, Hascoet H, Nicolas L. [Combinations of ivermectin and diethylcarbamazine for improved control of lymphatic filariasis]. Parasite. 1996;3 1:45-8; doi: 10.1051/parasite/1996031045. <https://www.parasite-journal.org/articles/parasite/pdf/1996/01/parasite1996031p45.pdf>.

12. Ismail MM, Weil GJ, Jayasinghe KS, Premaratne UN, Abeyewickreme W, Rajaratnam HN, et al. Prolonged clearance of microfilaraemia in patients with bancroftian filariasis after multiple high doses of ivermectin or diethylcarbamazine. Transactions of the Royal Society of Tropical Medicine and Hygiene. 1996;90 6:684-8.

13. Dreyer G, Addiss D, Noroes J, Amaral F, Rocha A, Coutinho A. Ultrasonographic assessment of the adulticidal efficacy of repeat high-dose ivermectin in bancroftian filariasis. Trop Med Int Health. 1996;1 4:427-32. <https://onlinelibrary.wiley.com/doi/pdf/10.1046/j.1365-3156.1996.d01-79.x>.

14. Dreyer G, Noroes J, Amaral F, Nen A, Medeiros Z, Coutinho A, et al. Direct assessment of the adulticidal efficacy of a single dose of ivermectin in bancroftian filariasis. Transactions of the Royal Society of Tropical Medicine and Hygiene. 1995;89 4:441-3.

15. Dreyer G, Coutinho A, Miranda D, Noroes J, Rizzo JA, Galdino E, et al. Treatment of bancroftian filariasis in Recife, Brazil: a two-year comparative study of the efficacy of single treatments with ivermectin or diethylcarbamazine. Transactions of the Royal Society of Tropical Medicine and Hygiene. 1995;89 1:98-102.

16. Moulia-Pelat JP, Nguyen LN, Glaziou P, Chanteau S, Ottesen EA, Cardines R, et al. Ivermectin plus diethylcarbamazine: an additive effect on early microfilarial clearance. The American journal of tropical medicine and hygiene. 1994;50 2:206-9.

17. Moulia-Pelat JP, Glaziou P, Nguyen LN, Chanteau S, Plichart R, Beylier I, et al. Ivermectin 400 micrograms/kg: long-term suppression of microfilariae in Bancroftian filariasis. Transactions of the Royal Society of Tropical Medicine and Hygiene. 1994;88 1:107-9.

18. Martin-Prevel Y, Cosnefroy JY, Tshipamba P, Ngari P, Chodakewitz JA, Pinder M. Tolerance and efficacy of single high-dose ivermectin for the treatment of loiasis. The American journal of tropical medicine and hygiene. 1993;48 2:186-92.

19. Kazura J, Greenberg J, Perry R, Weil G, Day K, Alpers M. Comparison of single-dose diethylcarbamazine and ivermectin for treatment of bancroftian filariasis in Papua New Guinea. The American journal of tropical medicine and hygiene. 1993;49 6:804-11.

20. Addiss DG, Eberhard ML, Lammie PJ, McNeeley MB, Lee SH, McNeeley DF, et al. Comparative efficacy of clearing-dose and single high-dose ivermectin and diethylcarbamazine against Wuchereria bancrofti microfilaremia. The American journal of tropical medicine and hygiene. 1993;48 2:178-85.

21. Cartel JL, Moulia-Pelat JP, Glaziou P, Nguyen LN, Chanteau S, Roux JF. Results of a safety trial on single-dose treatments with 400 mcg/kg of ivermectin in bancroftian filariasis. Trop Med Parasitol. 1992;43 4:263-6.
